# Challenges and prospects about the graphene role in the design of photoelectrodes for sunlight-driven water splitting

**DOI:** 10.1039/d0ra10176a

**Published:** 2021-04-16

**Authors:** Saulo A. Carminati, Ingrid Rodríguez-Gutiérrez, Andreia de Morais, Bruno L. da Silva, Mauricio A. Melo, Flavio L. Souza, Ana F. Nogueira

**Affiliations:** Institute of Chemistry, University of Campinas (UNICAMP) PO Box 6154 Campinas São Paulo 13083-970 Brazil flavio.souza@lnnano.cnpem.br anafla@unicamp.br; Centro de Ciências Naturais e Humanas, Universidade Federal do ABC (UFABC) Santo André São Paulo 09210-580 Brazil; Brazilian Nanotechnology National Laboratory (LNNano) Campinas São Paulo 13083-970 Brazil; Center for Information Technology Renato Archer (CTI Renato Archer) Rodovia D. Pedro I, km 143.6 13069-901 Campinas SP Brazil; Institute of Chemistry, Fluminense Federal University Outeiro de São João Batista, Campus do Valonguinho, Niterói Rio de Janeiro 24020-141 Brazil

## Abstract

Graphene and its derivatives have emerged as potential materials for several technological applications including sunlight-driven water splitting reactions. This review critically addresses the latest achievements concerning the use of graphene as a player in the design of hybrid-photoelectrodes for photoelectrochemical cells. Insights about the charge carrier dynamics of graphene-based photocatalysts which include metal oxides and non-metal oxide semiconductors are also discussed. The concepts underpinning the continued progress in the field of graphene/photoelectrodes, including different graphene structures, architecture as well as the possible mechanisms for hydrogen and oxygen reactions are also presented. Despite several reports having demonstrated the potential of graphene-based photocatalysts, the achieved performance remains far from the targeted benchmark efficiency for commercial application. This review also highlights the challenges and opportunities related to graphene application in photoelectrochemical cells for future directions in the field.

## Abstract 2

Graphene and its derivatives have emerged as potential materials for several technological application including sunlight-driven water splitting reactions.

## Graphical abstract

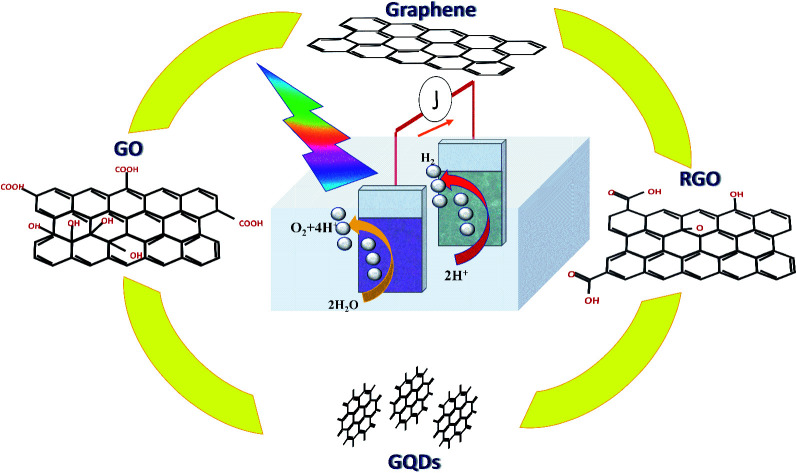

## 1 Introduction

Our society is facing a drastic environmental crisis associated with a significant dependence on non-renewable energy sources which release an uncontrollable amount of CO_2_ in the atmosphere. The scientific and industrial community efforts over the last decades have been spurred to develop carbon-free technologies. In this sense, artificial photosynthesis rises as one of the most elegant, sustainable, and renewable approaches to produce high energy density carriers, as molecules, driven by solar energy.

Several semiconducting materials such as metal oxides, nitrides or sulphides have been synthesized and their performance continuously evaluated as photoelectrodes. Materials such as TiO_2_, ZnO, α-Fe_2_O_3_, WO_3_, BiVO_4_, WO_3_, Cu_2_O, CdS, CZTS and MoS_2_ are the most studied materials associated with their potential for photoelectrochemical (PEC) applications. However, significative drawbacks present in those materials such as in TiO_2_,^1^ WO_3_ ^2^ and ZnO^3^ related to their large band gap enabling them to absorb only small portion of the solar spectrum have prevented the technology development. Another common limitation reported is the surface recombination that reduces the material performance and can be exemplified by BiVO_4_ ^4^ and α-Fe_2_O_3_.^5^ Cu-based materials have shown great performance in comparison with other cited system, however, it suffers with serious problem of chemical stability due to the photocorrosion process, shortening its lifetime as PEC component. To boost the PEC cell performance using photocatalysts, several strategies have been pursued, for instance, extending the absorption spectra towards infrared, improve charge carrier kinetics and increase the active sites at the surface including the doping with anions or cations,^6–8^ surface coupling with metals or semiconductors^9,10^ and film morphology design (nanoscaling).

In this sense, carbon-based materials in nanometric scale have called the attention of many researchers in the field due to their properties and potential to improve optical and electronic of those mentioned semiconductors. It has been proved that the coupling between a semiconductor with carbon-based material can efficiently enhance the PEC activity by suppressing recombination, extending the excitation wavelength, or increasing the surface-active sites.^11–13^ Since the pioneer report of graphene it become the most popular compound in the carbon-based family,^14^ mainly attributed to the presence of a singular sp^2^ carbon network that provides a privileged surface area, high conductivity and excellent electron mobility, ideal for applications in solar energy conversion.

In view of these highlighted characteristics, its application as photocatalyst modifier for helping them overcome the already discussed limitation on PEC cell was naturally incorporated in the field routine investigation. In fact, graphene and its derivatives such as reduced graphene oxide (rGO) enabled the photoelectrodes enhances the optical, conductive, and chemical properties of different materials, while the utilization of a single-layer graphene (SLG) optimizes their stability, electrical, and redox properties. Moreover, graphene quantum dots (GQDs) band gap and Fermi level can be modified as function of their particle size proving that their combination with semiconducting electrodes boost the efficient interfacial charge carrier transfer.

It is worth to mention that graphitic carbon nitride (g-C_3_N_4_) material has also attracted widespread attention due to low cost, appropriate electronic band structure (*E*_g_ ∼ 2.7 eV) corresponding to 460 nm in the visible range and excellent photochemical stability.^15^ However, only few studies have been dedicated to study its combination with graphene (or its derivatives) in photoelectrodes. The latest available reports show that graphene sheets can act as a support for the g-C_3_N_4_ growth as well as charge acceptor, thus improving the electronic transport of the film.^15–24^ Moreover, the graphene addition can lead to enhanced electrochemical active surface area, electron diffusion length and total exciton lifetime as recently demonstrated.^25^

Although, the research involving graphene-modified photoelectrode has reached the top in the last few years, not many insightful comprehensive review papers have been published. For this reason, this review summarizes and discusses the latest advances achieved in the field and highlights the existing limitations and how we could overcome them.^26–29^ This review paper focuses on the recent developments of graphene architectures coupled with different materials as photoelectrodes for photoelectrochemical water splitting reactions. This work contains a general introduction of the fundamentals of PEC devices and the main structure and properties of graphene and its derivatives suitable for solar energy applications. An insight into the role of graphene and its derivatives in boosting metal oxide photoanodes, photocathodes, heterojunction and non-metal semiconductors performances are carefully and systematically elucidated. Finally, future perspectives related to graphene addition as modifier in photoelectrodes for solar energy conversion are discussed. It is expected that our current review provides essential information in the field of solar energy conversion, and, simultaneously, offers a complete panorama (advantages and disadvantages) of the real effect of graphene and its derivatives as modifier for PEC applications.

### 1.1 PEC fundamental concepts

**Fig. 1:**
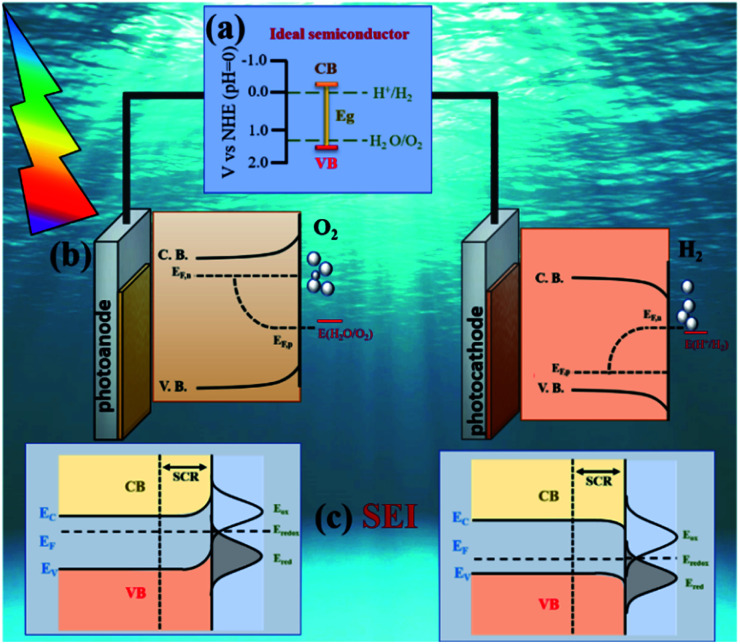
Schematic of the main design aspects for a PEC design: (a) semiconductor properties, including band gap (*E*_g_), band position edges and chemical stability; (b) energetics diagram for water photoelectrolysis during illumination. The quasi Fermi levels (*E*_F,n_ and *E*_F,p_) of the materials must be located above and below the water redox potentials for n-type and p type semiconductors respectively and (c) semiconductor electrolyte interface, influenced by the band bending and film structure. Adapted with permission from ref. 36 and 37. Copyright 2006, Elsevier and 2016, American Chemical Society.

Split water in the presence of a photocatalyst under sunlight to produce hydrogen represents a sustainable and attractive manner to store solar energy. A photoelectrochemical cell (PEC) is a device specially designed for this purpose in a very elegant way. In this set-up, two electrodes (at least one fabricated with a photoactive material) are able to capture the solar energy (photons) and transform it into the chemical energy required to activate the water redox reaction. Fig. 1 illustrates a basic design of a PEC cell where an n-type semiconductor photoanode and a p-type semiconductor photocathode are immersed in an electrolyte solution and connected through electric contact.

When a PEC cell is illuminated, electrons located in the semiconductor valence band (VB) are excited and promoted into the conduction band (CB) of the photocatalyst. A portion of these hole (h^+^)–electron (e^−^) pairs recombines, while another fraction is separated. After the separation is achieved, the electrons are collected by the back contact while the holes migrate to the photoanode surface to evolve the oxygen evolution reaction (OER); this phenomenon can be observed for the photocathodes in which the holes are collected while electrons are transferred to the interface to carry out the hydrogen evolution reaction (HER); when both are interconnected oxidation and reduction can be done simultaneously and can utilize light energy more efficiently. The mechanism will be explained in detail later in this section.

The eqn (1)–(3) show that the water splitting reaction require at least 1.23 V to take place, equivalent to 1.23 eV which is the theoretical minimum energy for this redox process. In order to accomplish this requirement, the semiconductor band gap (*E*_g_) must be larger than 1.23 eV but it needs to be small enough to absorb the major part of the solar spectrum, being the ideal value around 1.6–2.0 eV.^30–32^ It is important to mention that even if the semiconductor is capable to absorb efficiently sunlight, the band edge energies need to be positioned correctly to preference the OER and HER.

1 2H_2_O + 4h^+^ → O_2_ + 4H^+^ (OER)

2 2H^+^ + 2e^−^ → H_2_ (HER)

3 2H_2_O → O_2_ + 2H_2_ *E*_redox_ = 1.23 V

Ideally, the CB must be placed at higher energy than the HER potential while the VB must be located at lower energy than the OER potential; Fig. 1a describes the ideal band diagram of a semiconductor capable to split the water molecule. If both requirements are fulfilled, when the semiconductor is illuminated, it will absorb photons and electrons will be promoted from the VB to the CB to achieve the water splitting reaction at the interface.

Despite the fact that the semiconductor properties dictate their potential use in efficient solar water splitting PEC system, as the photoelectrodes are immersed in water, it is imperative to comprehend the thermodynamic and kinetic parameters involved in the solid–liquid junction which is created by the interaction between the semiconductor and the electrolyte, known as semiconductor–electrolyte interface (SEI). When the electrode is immersed into an electrolyte solution, charge transfer reactions occur until thermodynamic equilibrium is reached and the Fermi level energy (*E*_f_) of the semiconductor equals to the electrolyte redox potential.

At this moment, a charged interface is created, resulting in a potential drop at both semiconductor and electrolyte solution.

The *E*_f_ position with respect to the solution redox energy depends on the work function and excess positive or negative charge associated with the semiconductor electrode. In equilibrium, the band bending in case of n-type semiconductors results from an excess of positively charged donors resulting in an “upward” band bending, which is compensated by anion accumulation in the electrolyte solution at the surface. An opposite situation is observed in p-type semiconductors, where the *E*_f_ is below the redox potential of the electrolyte, implying a “downward” band bending^32–34^ as observed in Fig. 1c. The region where a band bending exists is known as space charge region (SCR) and its depth depends on the donor (acceptor) density, the semiconductor dielectric properties and the electric field. The flat band potential (*E*_fb_) is referred to the condition when a semiconductor in contact with another material is not polarized in the interfacial region. At this condition, the CB and VB are flat and no electric field acts on the charge carriers. More details about SEI and how the charge transfer processes at this interface are affected by film structure and preparation methods can be found elsewhere.^32,35^ It is important to note that the described phenomena occur under dark conditions; if the cell is illuminated, light induced processes will take place, and the electrode/electrolyte dynamics will be modified.

Under illumination, electron–hole pairs are photogenerated in the semiconductor. If the process is operated under open circuit conditions, the *E*_f_ increases due to the internal photovoltage. This process interrupts the thermodynamic equilibrium in the depletion region previously produced under dark conditions and the creation of a quasi-Fermi energy level is necessary to describe the electrochemical potential of the photo-generated electrons and holes (Fig. 1b). Those levels can be calculated according to the following equations:4
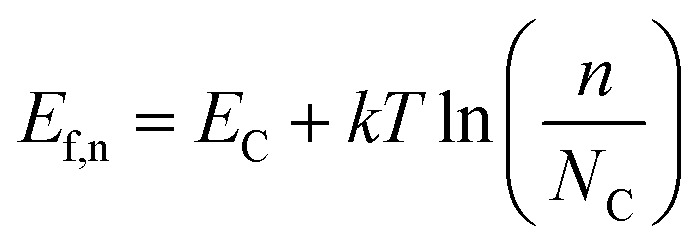
5
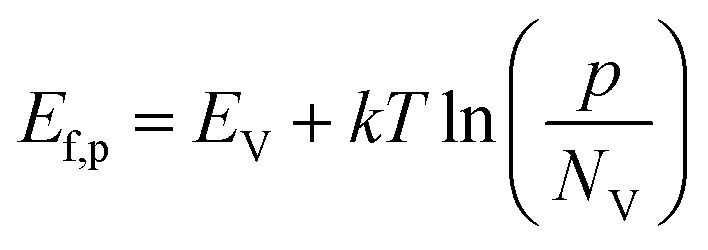
where *E*_C_ and *E*_V_ are the energies of the conduction and valance bands respectively, *n* and *p* the concentration of the majority charge carrier and *N*_C_ and *N*_V_ the density of states in the conduction and valance bands, respectively.

At low injection conditions, a change in the majority carrier concentration is not significant. The net current across the illuminated semiconductor/electrolyte interface is constituted by the current due to the reactions involving photogenerated minority carriers as well as majority carriers at the opposite side of the cell.

As explained previously, when the electrode is illuminated, charge carriers are photogenerated through light absorption. Due to the electric internal field generated at the semiconductor–electrolyte interface, the minority carriers are able to travel to the surface in a drift mechanism while the majority carriers diffuse to the bulk, process limited by the carrier diffusion length of the semiconductor. If carriers are generated in the bulk and the distance to the back contact is larger than the diffusion length, they will recombine. Charge carriers that are not lost by recombination, are transferred from the surface to the electrolyte, which is determined by thermodynamics and kinetics for charge transfer. To determine the efficiency of the system, it is necessary to employ the concept described by eqn (6). The applied bias photon-to-current efficiency (ABPE) is a parameter estimated from the current–potential curves under light condition as follows:6
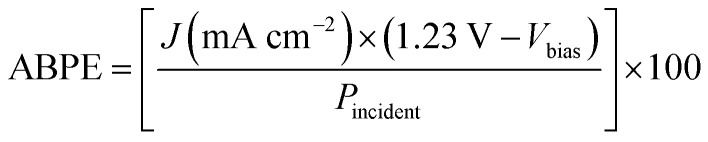
where *V*_bias_ is the bias applied between the working electrode and counter electrode, *P*_incident_ is the light power using an AM 1.5 filter and *J* is the electrode current density.

Moreover, the incident photon-to-current efficiency (IPCE) is a measure of the ratio of the photocurrent *versus* the rate of incident photons as a function of wavelength:7
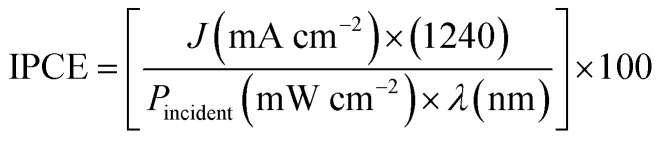


These two analyses, ABPE and IPCE, were proposed in literature as the most reliable way to compare performances of materials designed by different synthesis methodology.

Another alternative to calculate the total efficiency of solar radiant energy transformed into chemical hydrogen energy by a parameter known as solar to hydrogen efficiency (*η*_STH_), described by eqn (8):8
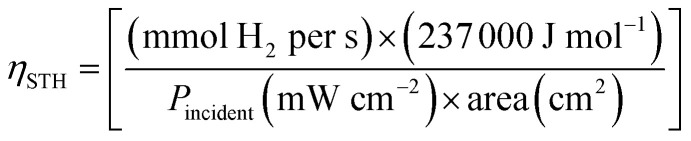
where mmol H_2_ per s corresponds to the H_2_ rate production and *P*_incident_ the total incident light power.

Over the past decades, effort has been devoted to design new materials or modify the existing materials to increase PEC device efficiency as described by these equations. Despite certain progress was reached, the predicted benchmark efficiency (over 10%) and stability for commercial application still far to be achieved. In recent years, graphene appeared as a promising material with positive impact when added during the fabrication of photoelectrodes. However, the role of graphene and how the properties are improved remain unclear. The next section will emphasize the main graphene properties which make it suitable for certain applications before introducing a discussion regarding its role in the photoelectrodes in PEC.

### 1.2 Graphene

Graphene is a metal-free material formed by a 2D carbon monolayer with a closely packed hexagonal lattice structure. Compared to graphite and carbon nanotubes, graphene exhibits much greater surface area and exceptional conductivity making it a potential candidate for different uses. Graphene has been reported in several applications such as a biosensors,^38^ gas sensors,^8^ electronic device design,^39^ transistors, fuel cells,^40^ photovoltaic devices,^41^ photocatalytic hydrogen generation, *etc.*

Graphene is composed by sp^2^ hybridized carbons with a band gap nearly to zero. The electrical conductivity of graphene is related to its carbon network, which is 60 times higher than carbon nanotubes conductivity.^39^ Graphene also possesses a high density of surface-active sites and it is thermally stable.^42,43^ Contemplating its remarkable electron mobility, conductivity and stability, graphene is an excellent candidate to improve the efficiency of PEC systems. It has been shown that the insertion of graphene helps to improve the charge separation and increase the electrode surface area. In parallel, the new interface between the graphene and the semiconductor facilitates the hole/electron extraction, enhancing charge transfer reactions. Moreover, graphene, employed as a scaffold, creates a 2D conductive support path for charge transport and collection to enhance the electron transport properties of semiconductors.^26,44^

Since the graphene owns a higher work function compared to other semiconductors, it can act as an electron bridge to accelerate the electron transfer from the semiconductor CB to graphene, achieving a better charge separation.^44,45^ It is worth mentioning that some reports have demonstrated that chemical bond formation and the Schottky junction between the semiconductor and the graphene can modify the semiconductor band gap and enhance the light absorption.^46,47^

However, regardless of its benefits, graphene poor dispersibility significantly reduce its electrical conductivity, surface area, optical transparency and diminish the mass transfer kinetics.^48–50^ Due to this contrast, graphene derivatives represent an interesting and challenging field to be studied.

One of the main challenges in the synthesis and application of graphene sheets is to overcome the strong cohesive van der Waals energy of the p-stacked layers in graphite.^51^ Usually, graphene and its derivatives are synthesized *via* graphene oxide (GO) which can be obtained easily by graphite oxidation and subsequently exfoliation.^52^ GO consists of a graphene structure modified with different functional oxygen groups such as carboxylic, hydroxyl and epoxide which offer some attractive advantages for photoactive materials.^50^

**Fig. 2:**
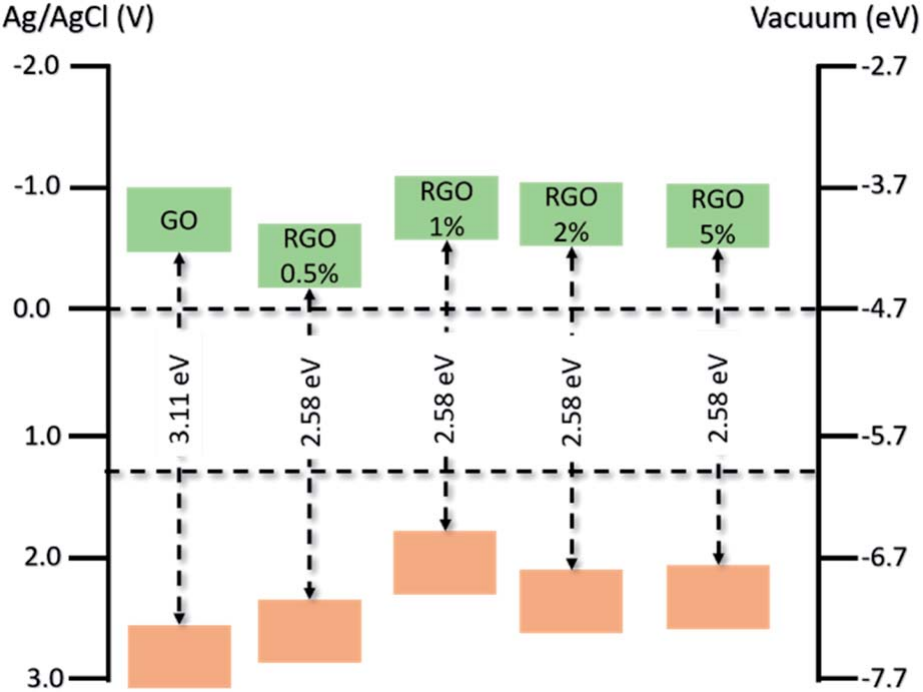
Energetic energy level diagram of rGO and GO/rGO electrodes at different GO wt%. The green and orange rectangles represent the CB and VB respectively. Adapted from ref. 56 with permission. Copyright 2019, Springer Nature.

Structurally, GO is an electronically hybrid material that features both conducting p states of sp^2^ carbon sites and a large bandgap between the s-states of its sp^3^-bonded carbons.^53,54^ This sp^2^/sp^3^ ratio can be modified by chemical reduction in order to produce reduced graphene oxide (rGO) which has brought the attention due to its remarkable conductivity and uniform incorporation in semiconductor electrodes. It has been proved that GO is able to drive the water splitting reaction by itself due to the band gap adjustment as a function of its oxidation degree.^55^ In fact, a recent work shows that an rGO/GO electrode achieves an enhanced response compared to GO, which the authors relate to the band gap tunning (displayed in Fig. 2), the faster electron acceptor capability of rGO and the electronic band structure of GO.^56^ Unfortunately, its response is still in order of microamperes and the system needs to be further studied.

As modifier, rGO offers a distinct advantage of being chemically obtained through the cheap, simple and well-established Hummer method compared to pure SLG, which is synthesized by chemical vapor deposition (CVD) technique, a more expensive method. Different reports have shown that rGO improves the optical, conductive, and chemical properties of different photoanodes and photocathodes.

Another graphene derivate that is drawing a lot of attention are the graphene quantum dots (GQDs). The quantum-confinement and edge effects^28,57^ associated with their particle size, usually less than 10 nm, provide highly active reaction sites at the electrode. Additionally, GQDs have demonstrated excellent chemical stability, low cytotoxicity and high luminescence.^28^ One of the main advantages of GQDs is related to their electronic structure which can be modulated according to the particle size, conjugating them with polyaromatic rings to enlarge the π-conjugated sp^2^-carbon network or by introducing an intermediate n-orbital between π and π* orbitals *via* functionalization with electron-donating chemical groups.^58^ It has been shown that GQDs modification in TiO_2_ electrodes decreases the system impedance and improves the charge transport kinetics.^57^ Indeed, in some cases GQDs have been employed to tune the band gap to enhance the light absorption in the visible spectrum.^59^

In contrast, the use of overlayers and underlayers to improve the charge carrier dynamics is a solid strategy that has been largely explored. The deposition of a single layer of graphene (SLG) has shown better properties than rGO or amorphous carbon for PEC applications. Due its transparency, SLG only absorbs 2% of the solar spectrum, not interfering with the electrode absorption. Moreover, this layer conserves the high mobility of the charge carriers in graphene.^60,61^ Furthermore, SLG is chemically inert in air and in electrochemical media.^62,63^ As other graphene derivatives, the SLG has demonstrated an intrinsic photoresponse.

Although all aforementioned, each photoelectrode material presents unique properties and characteristics that need to be analysed in detail prior graphene incorporation to maximize its efficiency. The next sections disclosure the performance of graphene/semiconductor photoelectrodes supported by different studies published in the last five years.

## 2 Metal oxides/graphene

For PEC applications, metal oxides semiconductors offer many advantages as photoelectrodes. When used as photoanodes, they are not susceptible to oxidation reactions. Additionally, the wide *E*_g_ range of these semiconductors brings the possibility of being used as absorber to arise higher efficiencies. In terms of costs, most of metal oxides are composed by earth abundant elements which make their fabrication affordable. Unfortunately, one of the main limitations is associated to their relatively poor carrier kinetics which is reflected on the photoelectrode performance. Over the last decades, several efforts have been focused on creating new methodologies for produce stable and efficient photoanodes/photocathodes. Recently graphene (and its derivatives) started to be considered an attractive material to incorporate into metal oxides systems to evolve the HER and OER reactions.

In this section, the recent developments of graphene/metal oxides as photoelectrode materials for water splitting will be highlighted. The graphene influence on the metal oxide photoanodes, photocathodes and heterojunction photoelectrodes reflected in different aspects (structurally, charge carrier dynamics and stability) during the water splitting process will be described as discussed in this section.

### 2.1 Photoanodes

Despite possessing unique and promising properties for water oxidation, many of metal oxides present intrinsic drawbacks^64^ that preclude their use as photoanodes by not reaching their maximum photocurrent density.^64,65^ It is noted that the key limiting factor of the poor PEC performance of many metal oxides is the high recombination rate of the charge carriers. Key strategies to address improvements for their use as stable and efficient photoanodes have been devoted. Researchers seek to include SLG, rGO,^66^ nitrogen-doped graphene^67^ and zero-dimensional GQDs^68^ as catalysts and/or supports in a wide range of photoanodes. In this section, a brief description of different graphene-based materials and their use as a “metal-free” carbon electrocatalyst materials^69^ on metal oxide photoanodes will be presented. Table 1 summarizes the most recent reports using graphene and its derivatives as a modifier in semiconductors for photoanode application.

**Table 1:** Recent published research in the past three years, using graphene or its derivatives as modifier in metal-oxides photoanodes for PEC characterization

Photoanode	Graphene derivative	Method for deposition	*J* (mA cm^−2^)	Measured potential	Electrolyte	Year (ref.)
Mo:BiVO_4_/G	rGO	Doctor blading	8.51	1.23 V_RHE_	0.1 M Na_2_SO_4_	2019 (ref. 80)
BiVO_4_/G/LDH	rGO	Electrodeposition	1.13	1.23 V_RHE_	KPi (pH 7)	2019 (ref. 79)
BiVO_4_/G/LDH	rGO	Hydrothermal	2.13	1.23 V_RHE_	0.1 M KPi	2017 (ref. 92)
BiVO_4_/G	rGO	Drop casting	0.13	0.8 V_Ag/AgCl_	0.1 M Na_2_SO_4_	2018 (ref. 93)
BiVO_4_/G	rGO	Drop casting	0.55	1.2 V_Ag/AgCl_	0.1 M Na_2_SO_4_	2018 (ref. 94)
BiVO_4_/G/NiFe	rGO	Spin coating	1.30	1.23 V_RHE_	0.5 M Na_2_SO_4_	2018 (ref. 95)
BiVO_4_/G/Co-Pi	GQDs	Electrophoresis	3.01	0.6 V_RHE_	0.1 M KPi	2019 (ref. 87)
BiVO_4_/LDH/G/CoPO_3_	pGO	Spin coating	4.45	1.2 V_RHE_	1 M potassium borate	2018 (ref. 96)
Co–N/P-GC-G/Fe_2_O_3_	Flexible exfoliated graphene	Spin coating	2.15	1.23 V_RHE_	1 M KOH	2017 (ref. 97)
Fe_2_O_3_/G	SLG	CVD	0.75	1.23 V_RHE_	1 M NaOH	2018 (ref. 74)
Fe_2_O_3_/G	rGO	Dip coating	0.88	1.23 V_RHE_	1 M NaOH	2017 (ref. 98)
Fe_2_O_3_/G	SLG	CVD	1.64	1.23 V_RHE_	1 M NaOH	2019 (ref. 70)
FeNiOOH/Fe_2_O_3_/G	Nanocarbon	Spray-coating	∼2.0	1.45 V_RHE_	1 M NaOH	2020 (ref. 99)
Fe_2_O_3_/G/Ag	rGO	Hydrothermal	0.72	1.23 V_RHE_	1 M KOH	2018 (ref. 100)
Ag–TiO_2_/G	rGO	Anodization	0.98	1 V_Ag/AgCl_	0.5 M Na_2_SO_4_	2019 (ref. 76)
TiO_2_–G	rGO	Drop casting	1.44	1 V_Ag/AgCl_	1 M KOH	2017 (ref. 101)
TiO_2_/G	GQDs	Spin coating	6.35	0.5 V_Ag/AgCl_	0.1 M Na_2_S	2017 (ref. 88)
TiO_2_/G	SNGQDs	Spin coating	0.85	0.5 V_Ag/AgCl_	1 M Na_2_SO_4_	2018 (ref. 85)
TiO_2_@Ag/G	GQDs	Layer-by-layer	∼0.6	1 V_Ag/AgCl_	0.5 M Na_2_SO_4_	2018 (ref. 102)
S–TiO_2_/G	S-doped rGO	Drop casting	3.36	1 V_Ag/AgCl_	1 M KOH	2018 (ref. 86)
TiO_2_/G	GQDs	Spin coating	0.26	1.23 V RHE	0.5 M Na_2_SO_4_	2020 (ref. 103)
ZnO NWs/G	N-doped GQDs	Layer-by-layer assembly	0.6	1.0 V_Ag/AgCl_	0.5 M Na_2_SO_4_	2016 (ref. 104)
ZnO/G	rGO	Electrochemical reduction	1.8	0.7 V_Ag/AgCl_	0.1 M KOH	2017 (ref. 105)
ZnO	rGO	Sol–gel dip coating	1.02	1.5 V_Ag/AgCl_	0.5 M Na_2_SO_4_	2018 (ref. 106)
WO_3_/G	rGO	Doctor blading	2.85	0.6 V_Ag/AgCl_	0.5 M Na_2_SO_4_	2017 (ref. 107)
CSA–PANI–WO_3_/G	rGO	Spin coating	1.54	1.23 V_RHE_	0.1 M Na_2_SO_4_	2020 (ref. 108)
WO_3_/silane/G	GO	Dip coating	1.25	1.23 V_RHE_	0.5 M Na_2_SO_4_	2018 (ref. 109)
WO_3_/G	rGO	Hydrothermal	750^a^	0 V_SCE_	0.05 M Na_2_SO_4_	2019 (ref. 110)

a Photocurrent value in the range of nA cm^−2^.

The incorporation of graphene with no or negligible defects in the form of a large area sheet is highly required to maintain or enhance its electrical conductivity, electron mobility and carrier density.^70^ In this way, SLG has high transparency (97.7%) at visible light (*λ* = 550 nm); the Fermi level band alignment between SLG and the photoactive material makes it convenient for use in photoelectrochemical applications.^71^ It is expected that the SLG presence would not lead to a change in the photoanode optical properties due to its very thin layer and high transparency.^72^ Since α-Fe_2_O_3_ typically suffer from undesirable surface states,^73^ the addition of SLG as a passivation layer leads to an impressive improvement in the PEC performance of hematite photoanodes. The passivation effect of the surface states after SLG incorporation on the top of the photoanode, resulted in an increased lifetime of the photogenerated holes.^70^ Additionally, as a consequence of the strong interaction between SLG and the photoanode, it was observed an efficient charge carrier separation and better hole accumulation at the photoanode surface,^70,74^ increasing the water oxidation efficiency.^70^ Although much higher photocurrent can be obtained after the deposition of SLG on the top of metal-oxides films, it is important to highlight that in this case different methods for SLG deposition should be taken into account for better interaction of the semiconductor with the graphene material, in such a way that not only the photoanode surface would be benefited by the SLG presence, but the whole composite.

Differently of SLG, the reduced graphene oxide (rGO) can lead to a significant improvement in the light absorption upon its addition on photoelectrocatalyst system. In this trend, the incorporation of rGO has been widely explored.^66^ It has been shown that when incorporated, rGO can broaden the absorption spectrum of many metal oxides that poorly absorb visible light.^75^ In general, it has been discussed that this may occur due to the orthogonalization of light in porous structure of carbon materials. The high porosity of rGO-based materials can lead to multiple reflection of light inside the film, improving the light absorption.^76,77^

The two-dimensional rGO nanosheets present high electron mobility and π-electron conjugation.^78^ Besides the light absorbance improvements, owing to its excellent electronic properties, the presence of rGO on top of the photoanodes can lead to an increase of the charge separation efficiency. As a consequence, a lower bulk recombination is obtained, which makes rGO as an effective electron capturing and transferring material.^79,80^ The onset potential shift suggest that the role of rGO is to increase the carrier transport and electron collection efficiency.

The application of rGO with plasmonic nanoparticles in order to enhance the PEC performance of conventional semiconductors has also been reported,^76^ in which a portion of hot electrons generated in metal nanoparticles can be accepted and shuttled *via* rGO sheets either by UV or visible light, minimizing charge recombination. Despite the incorporation of rGO has provided excellent improvements in photocurrent density of many graphene-based photoanodes, this material still contains a considerable amount of heteroatoms,^81^ as well as an incomplete restoration of a perfect carbon lattice,^82^ turning other graphene derivatives more attractive.

Besides the simple addition of graphene aiming better PEC performances by passivation effect, another approach is to make graphene properties tuneable.^83^ Graphene electronic properties can be tuned by doping it with heteroatoms, opening a new venue to further improve the performance of the metal oxides.^84^ By reacting epoxy, hydroxyl and carbonyl groups present in the rGO with molecules containing heteroatoms like N, P and S, these atoms can replace some of the carbon sites in the graphene framework structure, introducing defects. These intentionally added defects lead to improved chemical stability, as well as surface area and electron transfer.^84,85^ By doping with heteroatoms, the doped-graphene material can be tailored to act as electron donor or acceptor, depending on the electronegativity of the dopant with respect to carbon atoms. Such defects are responsible for opening the *E*_g_ in graphene-based materials, offering additional fine tuning of the band alignment.

In this context, it has been shown that sulfur doping greatly broad the photoanode light absorption.^86^ The efficient visible light harvesting resulted in the improvement in photocurrent density. The enhancement was attributed to the increased separation rate of electron–hole pairs as well as electron transfer at the surface, leading to a strongly chemical coupling. As consequence, a negative shift in the onset potential which was attributed to the negatively shifted Fermi level was noticed. The resulted positively charged carbon atoms turned them able to adsorb OH^−^ species, resulting the more favourable O_2_ evolution at the photoanode surface.

Since the photocurrent density is described as a function of the absorbed photocurrent (*J*_abs_), efficiency of charge carrier separation (*η*_sep_) and injection (*η*_inj_), different types of graphene derivatives can lead to different PEC behaviours.^90^ This trend can be simplified in the following equation:

9 *J*_PEC_ = *J*_abs_ × *η*_sep_ × *η*_inj_

For instance, a certain method for graphene deposition targeting to enhance light absorption can lead to higher *J*_abs_ values, however, by increasing the film thickness, recombination sites can be formed at the grain boundaries or at multiple layers and consequently, *η*_sep_ and *η*_inj_ values may be negatively affected.

**Fig. 3:**
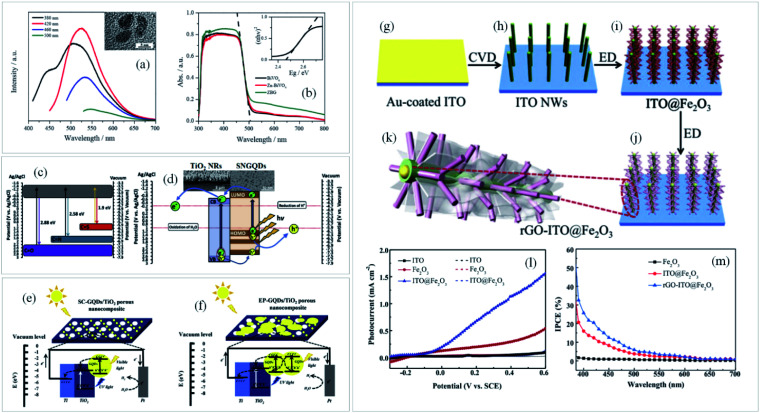
(a) PL emission spectra of GQDs at the excitation wavelength from 380 to 500 nm with their respective TEM images and the (b) UV-Vis spectra of BiVO_4_, Zn–BiVO_4_ and Zn-doped BiVO_4_/GQDs (ZBG). Reprinted with permission from ref. 87 Copyright 2019, Elsevier. (c) Energy levels of SNGQDs related to doping of oxygen, nitrogen and sulfur atoms and (d) energy diagram depicting charge transfer between TiO_2_ and SNGQDs. Reprinted with permission from ref. 85 Copyright 2018, Elsevier. Proposed mechanism for PEC water splitting by (e) spin-coated and (f) electrodeposited GQDs/TiO_2_ photoanodes. Reprinted with permission from ref. 88 Copyright 2017, Elsevier. Schematic illustration of (g–k) rGO-ITO@Fe_2_O_3_ photoanode fabrication process, (l) photocurrent density × applied potential and (m) IPCE plots of ITO, ITO@Fe_2_O_3_ and rGO-ITO@Fe_2_O_3_ photoanodes. Reprinted with permission from ref. 89 Copyright 2016, Elsevier.

It is worth mentioning that the architecture of the metal oxide in contact with graphene is equally important, leading to worse or better physicochemical interaction between the materials. In this circumstance,^89^ some nanostructured photoanodes (exemplified in Fig. 3g–m) decorated with rGO has shown an outstanding response related to the improved light absorption which activated the OER.^89^ In these cases, nanostructures serve as high-speed transport path for photogenerated electrons and a light absorber while rGO acts as electron conduction and surface passivation layer. Consequently, an enhancement of *J*_abs_ and *η*_sep_ parameters contribute to improve the overall photocurrent density.

Another interesting graphene derivative for this approach is the use of GQDs, which are mono or few-layer graphene sheets with lateral dimensions of less than 100 nm.^68^ Their unique properties such as excellent up-conversion, quantum confinement with size dependent photoluminescence (PL), high aqueous solubility, robust chemical inertness and low cytotoxicity^91^ open up new avenues and shed light on a novel branch of advanced nanostructured materials for solar-driven water splitting. For instance, by modulating the emission wavelength of the GQDs and matching it with the absorption edge of the semiconductor (Fig. 3a and b), both light harvesting and IPCE can be greatly improved.^87^

Doping graphene quantum dots with sulfur or nitrogen (SNGQDs) can input novel valuable properties to graphene-based materials. By co-doping them with different atoms, SNGQDs can originate two or more excitation wavelength-independent PL and broad the absorption in the visible region of light with multiple absorption edges,^85^ turning them auspicious photosensitizers for effective PEC applications. Depending on the doping atom, different energy levels of the SNGQDs (Fig. 3c) can be obtained leading to better alignment with the oxidation and reduction energy levels of water splitting reaction (Fig. 3d).

Interestingly, it has been shown that depending on the deposition method, GQDs can also play different roles in the photoanode performance. For instance, authors employed two different methods^88^ to deposit GQDs on TiO_2_ photoanodes, which led to a considerable difference in charge transfer and PEC performance. By using spin coating as deposition method, GQDs behave as a photosensitizer donating electrons to the CB of the semiconductor. On the other hand, if the amount of GQDs deposited on a specific photoelectrode is not well controlled, as occurred when deposited by electrodeposition, their excess causes blockage of electron transfer, in a way that instead of facilitating electron transfer to the semiconductor conduction band (Fig. 3e), the photogenerated electrons in the GQDs obtain more probability to be recombined with holes (Fig. 3f).

This section described a wide range of properties enhanced by the addition of graphene-based materials on metal oxide photoanodes. We note that the key limiting factor of the poor PEC performance of many metal oxides is the high recombination rate of the charge carriers. Despite such improvements have been achieved, many questions are still opened. We believe that further studies using appropriate techniques to improve the understanding of the real role of graphene on the enhancement of photoanodes efficiencies towards water splitting are very important. Generally, it has been reported that when incorporated onto metal oxide photoanodes, graphene acts improving light absorption and charge transfer, which led to a decrease in the charge recombination rate at the semiconductor/electrolyte interface, enhancing the PEC performance.

Graphene and its derivatives lead to a wide range of photocurrent density, varying from μA to mA cm^−2^, and their performances may be related to the quality of the synthesized material as well as the method for preparation and deposition. Most of the papers describing the incorporation of graphene as an electrocatalyst, use rGO as the main material, and the reason relies in the facility to synthesize it, being a versatile platform for many different composites. In general, two main properties are observed to be enhanced after the incorporation of graphene and its derivatives on different metal oxide photoanodes, the light absorption and the charge transfer. All the others described, as photogenerated holes lifetime, charge separation and photostability, can be interpreted as a consequence of them.

### 2.2 Oxide-based photocathodes

In a photoelectrochemical cell, photocathodes must provide enough cathodic current to reduce protons and produce H_2_ in a stable aqueous environment.^111^ Even if PEC water reduction is energetically more favourable against water oxidation reaction, finding a suitable and electrochemically stable material to evolve the water reduction represent a more difficult task. For this reason, the available reports of oxide semiconductors as photocathodes are limited compared to photoanodes studies.

Copper-based materials fulfill the basic requirements to act as photocathode with special attention in literature dedicated to compounds as CuO,^112^ Cu_2_O,^113^ CuBi_2_O_4_,^12^ CuFe_2_O_4_ ^114^ and Ca_2_Fe_2_O_5_.^115^ Despite the progress achieved, these materials suffer critical drawbacks such as photocorrosion leading to a poor material stability followed by some electronic limitations usually associated with fast charge recombination. To mitigate those common issues and further optimize the photocathode activity, several strategies have been employed, including graphene insertion in different facets. In this chapter, the effect of graphene insertion into metal oxide photocathodes focusing on electronic structure, charge carrier kinetics and stability will be revised accordingly to meet the consensus and contradictions in the literature.

As stated in the last section, graphene can modify different material properties depending on the deposition technique and the chosen derivative, but how this insertion affects the photocathode electronic structure? Some studies^12,116–118^ have shown by XPS and Raman that the graphene or rGO addition into Cu based photocathodes allow the formation of Cu–O–C bonds, indicating a superior interaction and interfacial charge transfer between graphene and Cu compounds which reduce the electron density around the Cu material. It is worth mentioning that in these cases, graphene-based materials have been synthesized separately and subsequently added into the electrode by methods such as spin coating, drop casting or impregnation. In contrast, different behaviour was observed when the graphene layer is added by CVD: XPS studies did not suggest any chemical interaction among graphene and Cu_2_O; due to the Cu_2_O porosity, the CVD layer could not fully cover all photocathode surface.^119^

Despite Cu–O–C bonds have been successfully demonstrated by some authors, optical measurements are primordial to understand the role of graphene and its derivatives into the photocathode electronic structure. Absorption measurements of photocathodes modified by graphene,^116^ rGO,^117^ and NGQDs^120^ indicated that this modification increases the absorption range of the nanocomposites in the visible region but, in all circumstances, the band gap analysis showed that graphene based materials have not been incorporated into the lattice. However, in cases when a higher concentration of graphene (or its derivatives) is added, a competition of light absorption between graphene and the semiconductor and light blocking effects^12,116,121,122^ are noticeable.

**Fig. 4:**
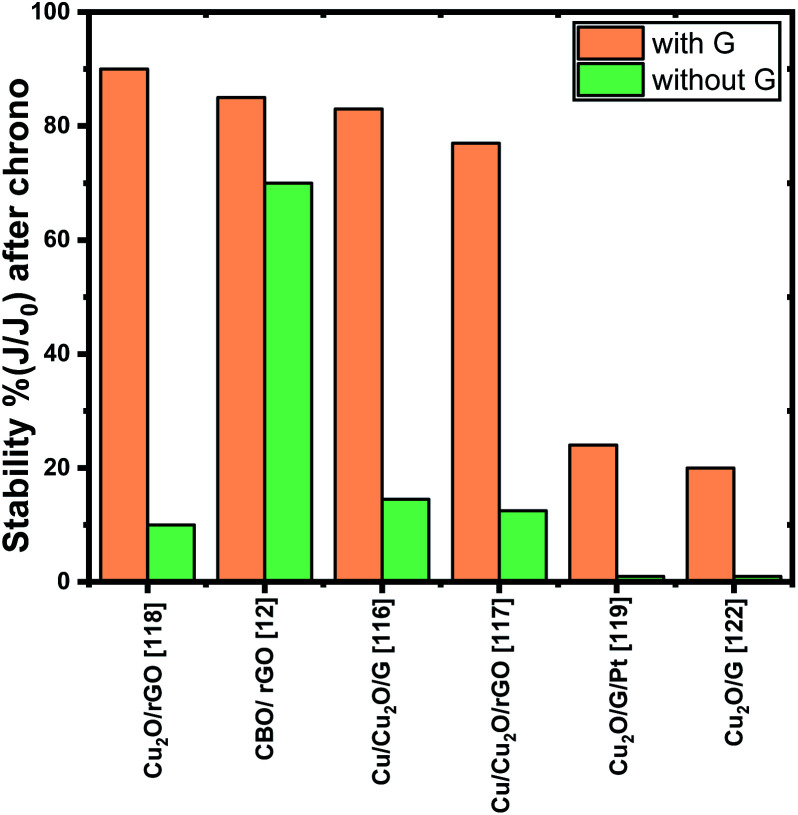
Stability percent of different systems before and after graphene (or graphene derivatives). All chronoamperometry measurements have been performed at 0 V *vs.* RHE at a given time.

Furthermore, to overcome the main drawback faced by Cu-based photocathodes, the photocorrosion, diverse strategies such as the passivation layers, crystal structure/morphology modification and cocatalyst addition have been employed to improve stability. Considering this approach, photocathode modification with graphene-based materials^116,118,119,121^ demonstrated to be a promising strategy to avoid the electrode photocorrosion. Since graphene and its derivatives can act as electron acceptors, they suppress the redox activity of the copper-based oxides photocathodes. Fig. 4 summarizes the photocurrent stability obtained by chronoamperometry measurements reported which is defined as the photocurrent density measured at the end of the test (*J*) divided by the photocurrent density at *t* = 0 s (*J*_0_); it is observed that, in all systems, the stability improved significantly compared to the bare system. Some authors^117–119^ affirm that the synergistic interaction between graphene and Cu photocathodes inhibit the catalyst self photocorrosion as well as improved the photocurrent density.

By analysing the stability (Fig. 4), two systems presented a lower enhancement (from 1% to ∼20%) when graphene is added; in both cases, graphene did not fully cover the Cu_2_O layer. For this reason, they deposited an additional layer of TiO_2_ ^119^ or amorphous molybdenum sulfide (a-MoS_*x*_)^122^ which improved the stability percent to 67% and 40% respectively. These studies bring the possibility of combining graphene with other semiconducting oxides to improve PEC systems. And in fact, the research in this field is pointing towards a combination of materials to succeeded in improved OER and HER reactions.

Although the chronoamperometric measurements have been performed at 0 V *vs.* RHE, the longest test data reported is 20 minutes which suggest the electrode photostability is not enough for scale-up. Even if several authors affirm that graphene electron mobility and its behaviour as electron acceptor boost the transfer of photogenerated electrons from photocathode to the electrolyte,^12,116,121^ it must be taken into account that Cu based materials by themselves can achieve remarkable performance during limited time and, for this reason, it is necessary to focus on resolve their stability drawbacks to then create strategies focused on charge transfer kinetics.

Despite all mentioned above provides valuable information to create new strategies for photocathode design, some issues are still elusive. Recently, Yu and collaborators^123^ determined that the GO modification on Cu_2_O/C/NiCoB films increases the photocathode surface area, suggesting that GO addition induce the formation of abundant catalytic sites facilitating the charge transfer during the water reduction reaction. Also, it is necessary to understand how the electrocatalytic effect of graphene and its derivates influences the charge carrier dynamics of the photocathodes which is observed in the *J*–*V* curves.^118,120^

Even if the quantity reported studies are still limited, the recent studies have demonstrated the importance of the graphene deposition technique; if an optimal deposition is achieved, graphene and its derivatives can create chemical bonds between the photocathode and their carbon network facilitating the charge carrier dynamics and the stability without blocking the photocathode light absorption. Although the clear improvement in the charge carrier dynamics that graphene brings to the photocathode performance, this fact is not fully understood and, in some cases, it is important to consider if graphene can be the best material to be used as passivation layer or can be combined with other semiconductors to enhance the metal oxide photocathode activity.

### 2.3 Heterojunctions

The possibility of improving the performance of water splitting photoelectrodes by the integration of two or more semiconductors with different optical and/or physical characteristics has been constantly reviewed.^124–129^ Several findings published to date confirm that drastic limitations of photoelectrodes, such as elevated charge carriers recombination rates and propensity of undergoing photocorrosion, can be mitigated by the proper connection of semiconductors, in which the formed intimate contact, referred as heterojunction, favours the spatial separation of photoexcited electrons and holes as a result of suitable band energy levels alignments.^124,125,130–132^ And in fact, we might consider that the combination of graphene with photoanodes and photocathodes discussed previously, is an heterojunction itself.

Since effective heterojunctions drive electrons and holes in opposite directions, especially under the influence of an applied bias, they minimize the surface/interface charge accumulation that provokes photocorrosion. This electric field built across the framework intensifies the charge carriers migration, excelling the electron–hole separation and, consequently, reducing their recombination at specific points of the crystalline structure.^124,133^

In general, graphene and its derivatives are incorporated in photoelectrode heterojunctions as solid-state mediators to intensify the charge shuttle between the components, due to their aforementioned inherent electronic properties. Besides, owing to its high specific surface area, graphene contributes to the photoelectrocatalytic reactions, as it provides extra adsorption and catalytic sites.^127,134^

Among the graphene-related rational designs recently employed in heterostructured water splitting photoelectrodes, rGO is the most recurrent because it presents striking advantages over pure graphene and GQD, as mentioned before.^125^ Furthermore, compared to GO, rGO possess a reduced number of oxygen-containing groups that can act as deep recombination centres.

In fact, based on evidences from recent publications, this balanced content of pendent functional groups on the reduced graphene oxide layers is the responsible for the effective linking between the semiconductors in heterojunctioned photoelectrodes, resulting in the broader usage of rGO as mediator compared to the other graphene-like structures. As the connected semiconductors also contain reactive surface functional groups, they interact with those pendent on the rGO sheets, creating bonds that ensure an effective contact between the heterostructure components.

The lack of functional groups in pure graphene tends to preclude a stable and concomitant connection of the graphene sheets with two semiconductors that might possess very distinct surface properties. On the other side, non-reduced GO and graphene quantum dots have higher densities of these oxygenated functional groups. As these groups act as charge carrier trap centres, they may impair the charge transport within the layer and, consequently, the charge shuttle between the photocatalysts and the overall photocatalytic efficiency.^89,125,134–136^

As a consequence, most of the research investigations employ effective routes in which the connection of the semiconductors is guaranteed by their interaction with GO containing high density of oxygenated groups, followed by a reduction procedure to eliminate the excess of these recombination centres.

**Fig. 5:**
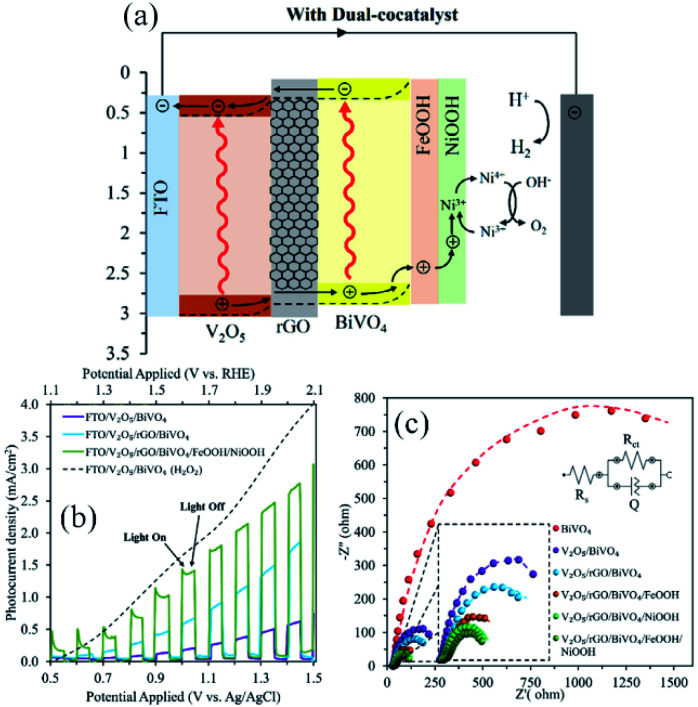
The application of rGO as the charge carrier mediator in the V_2_O_5_/BiVO_4_ heterojunction. (a) The energy diagram depicting different components of the photoanode, including rGO layer; (b) photocurrent density *versus* applied potential curves and (c) Nyquist plots of EIS for the electrodes prepared in this study. Reprinted with permission from ref. 140 Copyright 2020, Elsevier.

As a recent showcase, this strategy was adopted for a water splitting photoanode in which rGO was employed as the solid-state charge carrier mediator two oxide semiconductors (SC/rGO/SC).^79,92,132,137–139^ A clear example is schemed in Fig. 5a–c ^140^ where it is noticeable that the addition of rGO had a paramount contribution to the activity of the photoanode, as the charge transfer at the graphene-free interface was hindered by recombination centres at the intrinsically discontinued heterojunction interface. In contact with the oxide semiconductor, rGO nanosheet creates a Schottky junction which favours the transport of charge carriers from one oxide semiconductor to the other, lessening the recombination of electron–hole pairs.^125^ Also, it has been suggested that graphene layer reduces the interfacial charge transfer resistance.^141^ Moreover, some authors affirms that rGO tune the electrical connection between both semiconductors.^142^

Heterojunctions formed by earth abundant non-oxide semiconductors can also be improved by the rGO incorporation as a bridged solid-state mediator, forming type II heterostructure.^143–145^

**Fig. 6:**
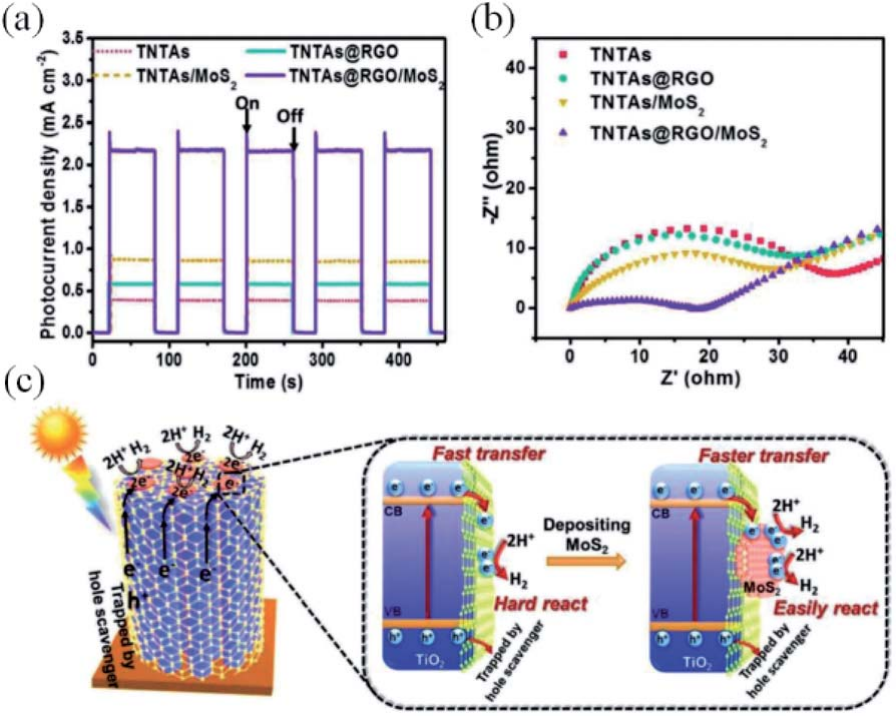
Results set for a ternary system containing MoS_2_ immobilized on a rGO-wrapped TiO_2_ nanotube array, applied as an efficient photoelectrode for water splitting. (a) Transient photocurrent responses, (b) EIS Nyquist plots of the prepared devices, and (c) schematic energy diagrams depicting the charge transfer dynamics of the photoelectrode under operation. Reprinted with permission from ref. 144 Copyright 2018, Wiley-VCH.

Especially for chalcogenides-based photoelectrodes, photoinstability is the primary disadvantage compared to metal oxides. Chalcogenides possess lower band gap values due to their energetic valence band edges, however their application as photoelectrodes is hindered by the strong photocorrosion tendency. This limitation can then be alleviated by a fast charge carriers extraction that might be accomplished by an adequate coupling with rGO.^125,143,145,146^ As an example, a substantial boost in the photocurrent was observed for the TiO_2_/rGO/MoS_2_ photocathode (Fig. 6a) compared to the binary TiO_2_/rGO and TiO_2_/MoS_2_ systems. A similar behaviour is observed in the Nyquist plots (Fig. 6b), which indicate that the charge transport resistance is much lower for TiO_2_/rGO/MoS_2_.^144^ An interesting point of debate raised by this work is that only a slight activity improvement took place when the single TiO_2_ film was solely wrapped by rGO layers due to the blockage of the TiO_2_ active sites for water reduction by rGO indicating its poor activity as cocatalyst. On the other hand, extra catalytically active sites are included by the attachment of MoS_2_ to the TiO_2_/rGO electrode, while rGO guarantees the efficient charge transfer between TiO_2_ and MoS_2_ (Fig. 6c).

This study highlights the importance of a following a rational design for the addition of graphene layers in a photoelectrode, as they may occlude important catalyst sites. An erroneous approach might offset the benefits brought by graphene in the charge transfer/separation.

**Fig. 7:**
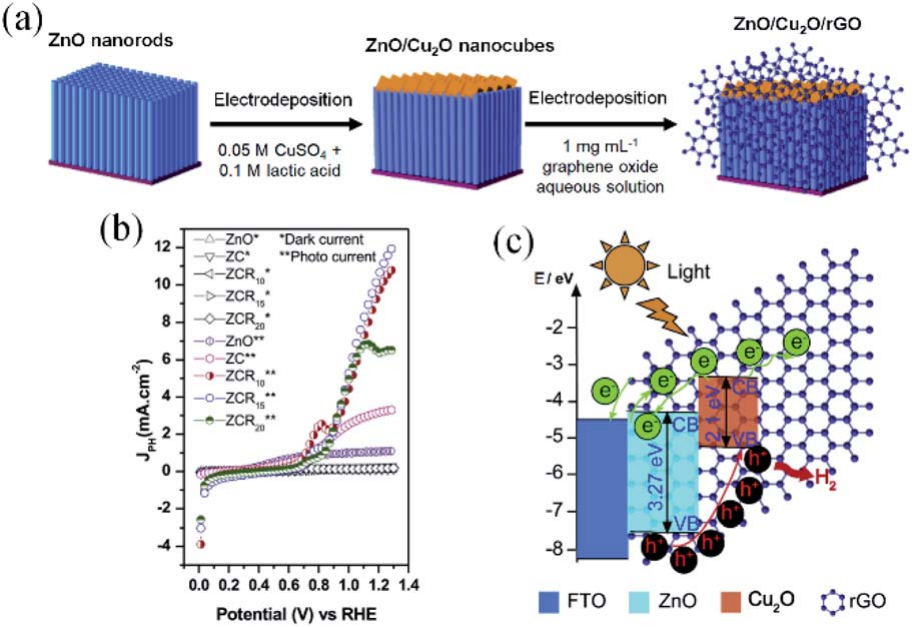
Results and schemes describing the synthesis and energy diagram of the ZnO/Cu_2_O/rGO heterostructured photoelectrode. (a) Scheme depicting the steps used for the photoelectrode preparation, (b) linear sweep voltammetry and (c) schematic energy diagram representing the charge mobility within the heterostructures under water splitting operation. Reprinted with permission from ref. 137 Copyright 2019, Elsevier.

By developing a different configuration for the inclusion of rGO in a type II heterojunction, Hou and co-workers^137^ were able to achieve an impressive photocurrent enhancement for a ZnO/Cu_2_O heterostructure from 3.22 to 10.11 mA cm^−2^ at 1.23 V_RHE_ (AM 1.5G). To design the photoelectrode, an intimate contact between ZnO nanorods (NRs) and Cu_2_O nanocubes was created, then rGO sheets wrapped the whole set as cocatalyst (Fig. 7a and b). The authors proposed that rGO sheets are responsible for the direct transport of photoexcited electrons from the heterojunction to the substrate. Due to its higher work function, rGO promoted directional electron transfer by forming Schottky junctions with both semiconductors.^125^ Meanwhile, the photoholes generated at ZnO are injected into Cu_2_O, inducing an effective electron–hole separation. These inferences are represented by the energy diagram in Fig. 7c.^125,143^

It is clear that the performance improvement achieved in this case results from a combination of the high crystallinity of the ZnO and Cu_2_O building blocks, the broad light absorption by Cu_2_O and the electric field induced by the p–n junction. However, rGO incorporation has a key role in the efficiency, as besides favoring a faster charge carrier diffusion, it acts in the passivation of Cu_2_O nanocubes, by preventing the direct contact with the electrolyte. Once again, it is important to highlight that the strong bonding between the semiconductors and rGO provided by the electroreduction method was of paramount importance for the performance and stability achieved for this photoelectrode. Additionally, rGO layer improves the Cu_2_O stability as discussed in Section 2.2 where it was observed that the wrapping graphene layers can also passivate the electrode by preventing the direct contact of Cu_2_O with the electrolyte.^129,137,138,147^

In summary, it has been demonstrated by the available literature that the photoelectrocatalytic performance of heterostructured photoelectrodes for water splitting can be enhanced by the incorporation of graphene at specific interfaces. As a rule of thumb, the substantial improvements observed in all cases are related to the high electrical conductivity of graphene sheets, which improve the charge carriers transfer across the heterojunction components.

Nonetheless, graphene-like species containing appropriate functional groups may strengthen the interfacial contact between semiconductors and increase the surface phase junction area, as it extends the interface between the semiconductors, intensifying the charge exchange.^141^ The photostability improvements of heterojunctions containing Cu_2_O and chalcogenides by the incorporation of graphene-like structures have also been reported. In all cases, the 2D network works as a rapid channel for the charge carriers transport, preventing charge accumulation at the unstable semiconductors.^79,144,148,149^

Some other points must be taken into account to further collect positive outcomes from the graphene-containing heterostructured photoelectrodes. The concomitant connection of the 2D sheets with both semiconductors is one important parameter that must be studied. This is a critical point that will affect the photoelectrode performance and stability, especially because of the harsh conditions usually used in the photoelectrochemical reactions. Thus, the composition and surface properties of the binding semiconductors must have good affinity with the functional groups pendent to the graphene-like sheets to ensure effective electronic contacts.

Apparently, reduced graphene oxide has been used in the great majority of the PEC heterojunctions due to the available oxygenated functional groups – such as carboxyl, hydroxyl and epoxy groups – that act as binding sites for the interaction with the reactive surface groups of the semiconductors that are connected.^136^ These features allow the creation of effective interfaces under mild synthetic conditions, such as the electrochemical reduction of GO, which was the method employed by most of the studies exposed in this review. Moreover, the electrical properties of rGO are tunable and adequate modifications might enhance the connectivity with another semiconductor surface to form heterojunctions.

**Fig. 8:**
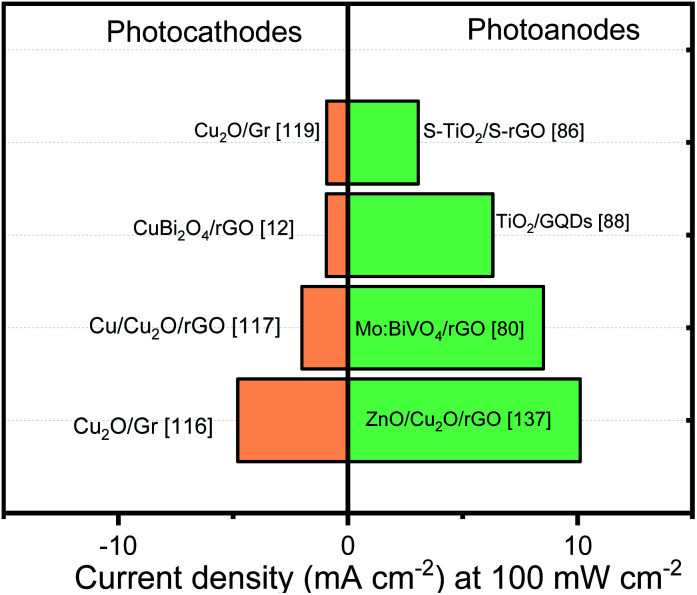
Photocurrent benchmark of graphene/oxides photoelectrodes. Data were extracted from various reports in the literature.

Fig. 8 exhibits the best photocurrent performance reported the last years oxides photoelectrodes modified with graphene. It can be noticed that even if graphene improves material stability, the photocurrent response is not close from the theoretical values or Cu_2_O best response (∼10 mA cm^−2^)^150^ where, additional to a protective layer, the formation of p–n junction and a cocatalyst deposition were indispensable for raising a promising performance.

In case of the photoanodes, the incorporation of rGO into different systems has shown the best results. The photocurrent values obtained by the rGO/heterojunction represent a good strategy to optimize the photoanodes. In contrast, although Mo:BiVO_4_/rGO nanocomposite performance can be interesting, the photocurrent values obtained by other methodologies are close from this benchmark.

Despite impressive photocurrent values have been obtained by the addition of many types of graphene and its derivatives in metal oxide semiconductors, the nature of the active sites remains elusive, leading to a superfluous comprehension of the role of the graphene. Therefore, future challenges should bring together *in situ* or *operando* with advanced techniques to amplify the comprehension of the mechanisms and species involved.

## 3 Non-oxide semiconductors/graphene

Non-oxide semiconductors are considered as attractive candidates for PEC solar fuel production due to their small band gap which permits capturing a wide range of the solar spectrum. Materials such as transition metal sulfides, selenides or silicon based photoelectrodes have been explored to evolve the OER or HER by themselves or combined with other semiconductors. However, during operation, few drawbacks associated with their physical, chemical and electronic properties have inhibited their performance to obtain high efficiencies. In this sense, graphene incorporation has been employed to overcome the problems associated with chemical and PEC corrosion, charge transport and separation deficits with remarkable results. In this section, the influence of graphene (and its derivatives) insertion on the PEC performance of non-oxide semiconductors in the past 5 years will be summarized. Due to the extensive nature of the non-oxide semiconductors and their peculiarities, this section has been further divided according their chemical structure.

### 3.1 Graphene-based transition metal chalcogenides (TMCs)

Transition metal chalcogenides (TMCs) are normally considered potential candidates for PEC water splitting. The VB of TMCs normally consists of 3p orbitals of S (or Se), which result in a more negative VB and a narrower *E*_g_ compared to metal oxides.^151^ However, the PEC performance using TMCs-based as photoelectrodes is still limited because of the fast charge carrier recombination. In addition, photocorrosion is actually a common problem for most TMCs.^152^ Besides a plethora of morphologies (*e.g.* nanowires,^153^ NRs^154,155^ and nanoflowers^154^), introduction of noble metal nanoparticles (*e.g.* Pt^156^ and Au^157^), formation of heterojunctions (*e.g.* TiO_2_/CdS^158^ and CdS/MoS_2_ (ref. 159)), the incorporation of graphene and/or its derivatives has also been widely used to improve electronic transport and decrease carrier recombination in these semiconductors. In order to gain a deeper understanding of the graphene-based TMCs, this section has been divided to analyze the binary transition metal sulfides, two-dimensional metal sulfides and ternary transition metal sulfides and selenides.

#### 3.1.1. Binary transition metal sulfides

Cadmium sulfide (CdS) is one of the most studied transition metal sulfides due to its appropriate band gap energy (*E*_g_ = 2.4 eV) and size-dependent electronic and optical properties.^159^ However, CdS is well-known by its instability and low photocurrent output. Different reports^160–163^ have demonstrated the graphene (and its derivatives) efficiency as electron acceptor and transporter. Upon light absorption, electrons from the CB of the semiconductor are quickly transferred to the graphene network, and then to the back contact. As observed for metal oxide photoelectrodes, an excess graphene content leads to a deterioration of the PEC performance, attributed to the “shielding effect”.^42^ Despite a photocatalytic and PEC improvement when graphene (and its derivatives) acts as electron acceptor, the enhanced photoresponse are still in μA cm^−2^. For this reason, additional to the graphene modification, some authors have decided to integrate the graphene/non-oxide semiconductor materials with other oxide semiconductors to overcome the charge collection deficit.

Due to its band gap, CdS been combined with wide band gap semiconductors (*e.g.* TiO_2_ and ZnO) to extend the absorption edge into visible light region and improve photoelectrochemical activity.^164,165^ As observed for metal oxide heterojunctions, graphene sheets (and its derivatives) can also be wrapped between two semiconductors to achieve better charge separation. Several reports^155,166–168^ have shown that a graphene interlayer (mostly rGO) between the semiconductor leads a photocurrent density improvement associated with a decrease in the recombination. However, as CdS acts as absorber layer and it is directly exposed to the electrolyte solution, the photocurrent decay of the systems can be related to Cd photocorrosion which has not been addressed by the authors.

For wide band gap transition metal sulfide semiconductors, graphene and its derivatives can also improve photogenerated carrier separation and transport. Zinc sulfide (ZnS) is a wide band gap semiconductor (*E*_g_ = 3.6 eV), which means that it cannot be photoexcited by visible light irradiation. A particular case where rGO is used as support for the growth of ZnS/Cd NRs open the possibility of designing new organized heterojunctions with attractive properties.^156^ Additionally to the photocurrent improvement associated with graphene as charge transfer mediator,^158,160,169^ the ZnS role as charge collector and the CdS absorption capability, the authors could not reproduce a similar pattern on the heterojunction without rGO addition.

Table 2 shows some examples of others photoelectrodes based on binary transition metal sulfides and graphene (or its derivatives) with their respective PEC performance. We can observe that there is a trend in the literature to synthesize graphene-based TMCs nanocomposites using chemical methods such as hydrothermal and solvothermal methods. Generally, these one-step methods require low-cost and simple equipment (*e.g.* autoclave and an oven) and can achieve a high-yield and large-scale production. During the hydrothermal synthesis, the GO, employed as precursor, is reduced into the reactor to obtain rGO which usually present structural defects, such as vacancies, heptagon and pentagon rings, edge effects and residual functional groups.^179^ Although these defects significantly affect electronic and chemical properties of the rGO sheets, the recent reports (Table 2) showed that this low cost methodology allows to design interesting heterostructures with higher photoresponses compared to other sophisticated methodologies needed to deposit graphene. On the other hand, the chemical methods permit to control several important experimental parameters. Consequently, a better interaction and control over the size and shape distributions of TMCs nanoparticles on the rGO sheets can also be obtained.^66^

**Table 2:** PEC performances of graphene-based binary transition metal sulfides photoelectrodes^a^

Photoanodes	Preparation method	Substrate	Light source	Electrolyte	Applied potential	Photocurrent density	Year (ref.)
Gr–CdS NPs	Chemical solution	FTO	300 W Xe arc lamp (100 mW cm^−2^)	0.5 M Na_2_SO_4_	1.0 V_RHE_	0.024 mA cm^−2^	2020 (ref. 170)
CdS NPs/N,S-co-doped rGO	Low temperature calcination	FTO	300 W Xe arc lamp (*λ* ≥ 420 nm, 100 mW cm^−2^)	0.5 M Na_2_SO_4_	0 V_Ag/AgCl_	0.010 mA cm^−2^	2018 (ref. 171)
CdS NPs/rGH	Hydrothermal	FTO	500 W Xe arc lamp (320–780 nm, 80 mW cm^−2^)	0.1 M Na_2_SO_4_	0 V_SCE_	0.10 mA cm^−2^	2018 (ref. 172)
CdS QDs/GQDs/TNTs	Solvothermal, ion-exchange reaction and sulfurization	FTO	300 W Xe arc lamp (*λ* ≥ 420 nm, 100 mW cm^−2^)	0.1 M Na_2_SO_4_	0 V_Ag/AgCl_	0.045 mA cm^−2^	2020 (ref. 158)
CdS core/Gr/TiO_2_ shell	Hydrothermal	FTO	150 W Xe arc lamp (AM 1.5G, 100 mW cm^−2^)	0.1 M Na_2_S	0 V_Ag/AgCl_	0.13 mA cm^−2^	2020 (ref. 173)
CdS NPs/NiS sheets/rGO	Solvothermal	FTO	300 W Xe arc lamp (*λ* ≥ 420 nm, 100 mW cm^−2^)	0.5 M Na_2_SO_4_	0 V_Ag/AgCl_	∼7.5 μA cm^−2^	2018 (ref. 167)
CdS–Sn_2_S_3_ clusters/rGO	Hydrothermal	GCE	300 W Xe arc lamp (*λ* ≥ 420 nm, 100 mW cm^−2^)	0.5 M Na_2_SO_4_	0 V_Ag/AgCl_	0.026 mA cm^−2^	2018 (ref. 168)
g-C_3_N_4_ NSs/CdS NRs/rGO	Wet-chemical	ITO	300 W Xe arc lamp (*λ* ≥ 420 nm, 100 mW cm^−2^)	0.1 M Na_2_SO_4_	0 V_Ag/AgCl_	0.38 μA cm^−2^	2017 (ref. 155)
ZnS nanospheres/rGO	Hydrothermal	GCE	110 W Xe arc lamp (*λ* ≥ 420 nm, 100 mW cm^−2^)	0.5 M Na_2_SO_4_	1.23 V_RHE_	1.1 mA cm^−2^	2018 (ref. 174)
ZnS NRs/N-doped Gr	Hydrothermal, thermal treatment and electrophoretic deposition	ITO	300 W Xe arc lamp (*λ* ≥ 420 nm, 100 mW cm^−2^)	0.1 M Na_2_S, 0.04 M Na_2_SO_3_ and 3 M NaCl	0 V_SCE_	5.2 μA cm^−2^	2018 (ref. 175)
ZnS/flower-like Gr	Hydrothermal and CVD	ITO	300 W Xe arc lamp (100 mW cm^−2^)	0.1 M Na_2_S, 0.040 M Na_2_SO_3_ and 3 M NaCl	0 V_Ag/AgCl_	∼9.8 μA cm^−2^	2017 (ref. 176)
ZnO NPs/ZnS NPs/rGO	Chemical solution	ITO	Mercury lamp	0.2 M NaOH	0 V_Ag/AgCl_	∼7.8 μA cm^−2^	2018 (ref. 177)
WO_3_ nanoplates/Sb_2_S_3_ NPs/Gr	Chemical bath deposition	FTO	300 W Xe arc lamp (AM 1.5G, 100 mW cm^−2^)	0.5 M Na_2_SO_4_	1.23 V_RHE_	1.2 mA cm^−2^	2017 (ref. 162)
rGO/Cu_2_S NRs/rGO/GO	SILAR	ITO	500 W Xe arc lamp (100 mW cm^−2^)	0.1 M Na_2_S	0.16 V_Ag/AgCl_	2.0 mA cm^−2^	2016 (ref. 178)

a Gr = graphene; rGH = rGO hydrogel; NPs = nanoparticles; NRs = nanorods; TNTs = titanate nanotubes; CoNHs = cobalt-based nanohybrids (Co_3_S_4_/CoS_2_); GCE = glass carbon electrode; CFP = carbon fiber paper; SILAR = successive ionic layer adsorption reaction.

#### 3.1.2. 2D transition metal sulfides

The combination of two-dimensional (2D) transition metal sulfide layers with graphene or its derivatives for photocatalysis and PEC applications has also been reported and some examples are summarized in Table 3. In these 2D transition metal sulfides, tin disulfide (SnS_2_), molybdenum disulfide (MoS_2_) and tungsten disulfide (WS_2_) are typical representatives. Benefited from their low cost, non-toxicity, large surface area, high stability and environmentally friendly characters, the layered transition metal disulfides with narrow *E*_g_ (∼2.1 eV) make them good semiconductors with visible-light-responsive ability.^180,181^ Although great achievements have been realized on 2D transition metal sulfide photoelectrodes, the incorporation of graphene or its derivatives can also promote the rapid transfer of carriers, inhibiting the electron–hole pairs recombination and extending the lifetime of photogenerated charge carriers.^180,181^

**Table 3:** PEC performances of graphene-based layered transition metal sulfides photoelectrodes^a^

Photoelectrodes (photoanode or photocathode)	Preparation method	Substrate	Light source	Electrolyte	Applied potential	Photocurrent density	Year (ref.)
MoS_2_/Gr (photoanode)	Liquid exfoliation	FTO	150 W Xe arc lamp	0.1 M Na_2_SO_4_	0.5 V_Ag/AgCl_	0.051 mA cm^−2^	2018 (ref. 192)
GO/MoS_2_ QDs (photoanode)	Hydrothermal	ITO	300 W Xe arc lamp (*λ* ≥ 420 nm, 100 mW cm^−2^)	0.5 M H_2_SO_4_	0.4 V_RHE_	0.090 mA cm^−2^	2018 (ref. 193)
3D TiO_2_ NPs/MoS_2_/Gr-aerogel (photoanode)	Hydrothermal	ITO	350 W Xe arc lamp (120 mW cm^−2^)	0.5 M Na_2_SO_4_	0.6 V_Ag/AgCl_	105 μA cm^−2^	2019 (ref. 184)
AgInZnS nanospheres/MoS_2_–GO (photoanode)	Hydrothermal	FTO	300 W Xe arc lamp (*λ* ≥ 420 nm, 100 mW cm^−2^)	0.5 M Na_2_SO_4_	0 V_Ag/AgCl_	0.17 μA cm^−2^	2018 (ref. 190)
CuInZnS nanospheres/MoS_2_–GO (photoanode)	Hydrothermal	FTO	300 W Xe arc lamp (*λ* ≥ 420 nm, 100 mW cm^−2^)	0.5 M Na_2_SO_4_	0 V_Ag/AgCl_	1.12 μA cm^−2^	2018 (ref. 189)
NiCo_2_O_4_ NPs/MoS_2_–GO (photoanode)	Hydrothermal	ITO	150 W Xe arc lamp (100 mW cm^−2^)	1 M KOH	0.8 V_Ag/AgCl_	5.36 mA cm^−2^	2018 (ref. 188)
g-C_3_N_4_/Gr/MoS_2_ (photoanode)	Hydrothermal	FTO	Xe arc lamp (100 mW cm^−2^)	0.2 M Na_2_SO_4_	0 V_Ag/AgCl_	∼1.1 μA cm^−2^	2018 (ref. 194)
MoS_2_/rGO/Cd_0.6_Zn_0.4_S NPs (photoanode)	Solvothermal	FTO	300 W Xe arc lamp (*λ* ≥ 420 nm, 100 mW cm^−2^)	0.3 M Na_2_S and 0.3 M Na_2_SO_3_	0 V_Ag/AgCl_	∼5.0 μA cm^−2^	2018 (ref. 187)
MoS_2_/g-C_3_N_4_/GO (photoanode)	Ion exchange	ITO	450 W Xe arc lamp (AM 1.5G, 100 mW cm^−2^)	0.1 M KCl and 0.1 M Eu(NO_3_)_3_	0.4 V_RHE_	1.0 mA cm^−2^	2017 (ref. 143)
CdS NRs/Gr/MoS_2_ (photoanode)	Hydrothermal	FTO	300 W Xe arc lamp (100 mW cm^−2^)	0.1 M Na_2_SO_4_	0 V_Ag/AgCl_	0.013 mA cm^−2^	2017 (ref. 186)
α-Fe_2_O_3_ (photoanode) and g-MoS_2_@Gr (photocathode)	Mechanochemical	GCE	300 W Xe arc lamp (AM 1.5G, 100 mW cm^−2^)	0.5 M H_2_SO_4_	−0.365 V_Ag/AgCl_	1.75 mA cm^−2^	2019 (ref. 195)
SnS NSs/ErGO (photocathode)	Electrodeposition	FTO	Red LED light (20 mW cm^−2^)	0.1 M K_2_HPO_4_ and 0.1 M KH_2_PO_4_	−0.6 V_Ag/AgCl_	−66 μA cm^−2^	2019 (ref. 196)

a Gr = graphene; ErGO = exfoliated reduced graphene oxide; NPs = nanoparticles; NSs = nanosheets; mpg-C_3_N_4_ = mesoporous graphitic carbon nitride; GCE = glassy carbon electrode.

MoS_2_ is also a typical layered transition-metal dichalcogenide with three stacked atomic layers (S–Mo–S) held together by van der Waals forces. This material has attracted the scientific community attention due to the presence of highly reactive edge sites showing efficient photocatalytic and PEC activities.^182^ In addition, 2D MoS_2_ nanosheets have appropriate *E*_g_ for solar absorption that can be tuned within the range 1.2–2.0 eV depending on thickness and lateral size.^160^ Similar to CdS, the MoS_2_ nanosheets can also extend the light absorption of other semiconductors (*e.g.* TiO_2_ and ZnO) in the visible region.^183,184^ Coupling graphene and its derivatives with MoS_2_ is also one of the strategies widely used to circumvent the problem of high charge carrier recombination rate.^159,185^ Ternary heterojunction nanocomposites composed by MoS_2_, graphene (or its derivatives) (CdS,^159,186^ g-C_3_N_4_,^143,182^ Cd_0.6_Zn_0.4_S,^187^ NiCo_2_O_4_,^188^ CuInZnS,^189^ AgInZnS,^190^ and Cu_2_ZnSnS_4_,^145^ ZnO^183^) have evidenced a similar trend: when graphene (or its derivatives) is added onto MoS_2_ no significative enhancement is observed; however, when MoS_2_/graphene is deposited on top of other semiconductor (SC/MoS_2_/graphene) an important photoresponse is noticeable. The authors attributed this enhancement to the electron transfer from the CB of ZnO to the CB of MoS_2_ and then to graphene. Unfortunately, some reports do not show the response of SC/MoS_2_ so it is complicated to define what part of this improvement is due to graphene insertion.

The edges of the layered MoS_2_ constitute the active sites for many important reactions.^185^ Numerous efforts have been devoted to maximize the exposure of active edge sites and improve the PEC performances. In this context, the graphene sheets (and its derivatives) can serve as a support for the growth of MoS_2_ and accelerate the interfacial electron transfer.^191^ Among the synthesis techniques, CVD offers unique advantages for achieving uniform graphene films on substrates in a controllable manner and with less structural defects. The edge-rich MoS_2_ grown on the edge-oriented 3D graphene glass synthesized by CVD can achieve the optimized charge transport along the 2D vector plane from MoS_2_ layers to graphene. In short, the 3D-graphene/E-MoS_2_ photoelectrode exhibited strong and broadband absorption, efficient exciton separation and good electron transport. In contrast with the metal oxide systems, the use of CVD technique to get a uniform substrate coating to promote the charge transport instead of covering a porous layer, represent a promising strategy to enhance the photoelectrode performance.

#### 3.1.3. Ternary transition metal sulfides and selenides

A new category known as ternary transition metal sulfides has also been widely used in PEC water splitting because these semiconductors exhibit high absorption coefficient over a wide spectral range. The use of two different metals allows access to the *E*_g_ not accessible to binary transition metal sulfides.^197,198^ It has been noticed that solvothermal synthesis that in some cases rGO insertion can shift the absorption edge of the sulfide and decrease the band gap.^198^ Additionally, the rGO avoid the formation of undesirable phases which inhibit the performance. Due to their excellent electrical conductivity, the rGO sheets also acted as electron collectors and transporters to efficiently suppress the recombination of photoinduced electron–hole pairs.

The development of research for novel transition metal sulfide nanomaterials and their graphene-based composites that do not contain acutely toxic metals, such as cadmium (Cd) and lead (Pb), has gained attention in recent years. There has been a slow but steady and conscious increase in the use of friendly environmentally materials. The ternary (ABS_2_) transition metal sulfide QDs (*e.g.* CuInS_2_ (ref. 199) and AgInS_2_ (ref. 200)) have been identified to be eco-friendly. Despite the authors demonstrated a photostability improvement and superior charge carrier kinetics when graphene is added, the photocurrent density is rather low, and this system cannot be considered yet as a serious candidate for PEC applications.

As the ternary (ABS_2_) transition metal sulfide, the ZnIn_2_S_4_ of the (AB_2_S_4_) family has attracted attention due to its narrow *E*_g_ (2.34–2.48 eV), high chemical stability and high surface area.^201,202^ Similar to the situation of other frequently studied semiconductors, the PEC performance of ZnIn_2_S_4_ can also be enhanced with the incorporation of graphene (and its derivatives).^203,204^ Analyzing the information in Table 4, the photocurrent densities are below the benchmark implying a great opportunity on the field. Indeed, the reports which showed a photocurrent density close to 1 mA cm^−2^,^205^ a hole scavenger was added to the electrolyte solution, indicating that the electrode is still facing hole injection deficiencies. Also, the high cost of indium prevents the large-scale use of these semiconductors in practical applications.

**Table 4:** PEC performances of graphene-based ternary transition metal sulfides photoelectrodes^a^

Photoanodes	Preparation method	Substrate	Light source	Electrolyte	Applied potential	Photocurrent density	Year (ref.)
ZnS QDs/CdIn_2_S_4_ NSs/rGO NSs	Solvothermal	GCE	300 W Xe arc lamp (*λ* ≥ 420 nm, 100 mW cm^−2^)	1 M Na_2_SO_4_	0 V_Ag/AgCl_	107 μA cm^−2^	2017 (ref. 214)
Flower-like ZnIn_2_S_4_ microspheres/g-C_3_N_4_ NSs/Gr	Hydrothermal	ITO	300 W Xe arc lamp (100 mW cm^−2^)	0.1 M Na_2_SO_4_	0 V_Ag/AgCl_	0.5 μA cm^−2^	2019 (ref. 201)
ZnIn_2_S_4_ NSs/rGO	Solvothermal and LAL	FTO	300 W Xe arc lamp (*λ* ≥ 400 nm, 80 mW cm^−2^)	0.5 M Na_2_SO_4_	0 V_Ag/AgCl_	∼1.7 μA cm^−2^	2019 (ref. 203)
Flower-like ZnIn_2_S_4_ microspheres/rGO	Microwave-assisted hydrothermal	ITO	300 W Xe arc lamp (100 mW cm^−2^)	0.4 M Na_2_SO_4_	0 V_Ag/AgCl_	18 μA cm^−2^	2017 (ref. 204)
Cd_0.6_Zn_0.4_S NRs/N-doped rGO	Solvothermal	ITO	300 W Xe arc lamp (*λ* ≥ 420 nm, 100 mW cm^−2^)	0.35 M Na_2_S and 0.25 M Na_2_SO_3_	0 V_RHE_	920 μA cm^−2^	2019 (ref. 163)
Zn_*x*_Cd_*y*_S NPs/GO/TiO_2_ NTs	Anodization and SILAR	Ti mesh	300 W Xe arc lamp (AM 1.5G, 100 mW cm^−2^)	0.1 M Na_2_SO_4_ and 25 vol% methanol	0 V_Ag/AgCl_	1.0 mA cm^−2^	2018 (ref. 205)
CuSbS_2_ QDs/TiO_2_ NWs/Gr	Wet chemical	FTO	300 W Xe arc lamp (AM 1.5G, 100 mW cm^−2^)	0.1 M Na_2_SO_4_	0.1 V_SCE_	287 μA cm^−2^	2019 (ref. 215)
TiO_2_ nanotubes/CdSe/GO (photoanode)	Potentiostatic anodic polarization and painting	Ni grid	500 W Xe arc lamp (16 mW cm^−2^)	1 M Na_2_S and 1 M KOH	−0.6 V_Ag/AgCl_	6 μA cm^−2^	2019 (ref. 207)
CdSe/graphene quantum dots (GQDs) (photoanode)	Hydrothermal	ITO	500 W Xe arc lamp (*λ* ≥ 420 nm, 100 mW cm^−2^)	1 M Na_2_S and 1 M Na_2_SO_3_	0 V_Ag/AgCl_	30.9 μA cm^−2^	2015 (ref. 216)
Cu(In,Ga)Se_2_/CdS/rGO/Pt (photocathode)	Co-sputtering and selenization	Mo-coated glass	300 W Xe arc lamp (*λ* ≥ 420 nm, 100 mW cm^−2^)	0.5 M Na_2_SO_4_ and 0.5 M Na_2_HPO_4_	0 V_RHE_	−22.4 mA cm^−2^	2018 (ref. 211)
α-Fe_2_O_3_/FeSe_2_@CoSe_2_/rGO (photoanode)	Solvothermal	GCE	300 W Xe arc lamp (*λ* ≥ 420 nm, 100 mW cm^−2^)	1 M KOH	1.4 V_RHE_	0.21 mA cm^−2^	2018 (ref. 208)
Graphene–Sb_2_Se_3_ (photocathode)	Electrodeposition	ITO	300 W Xe arc lamp (*λ* ≥ 420 nm, 100 mW cm^−2^)	0.5 M Na_2_SO_4_	0 V_RHE_	−0.65 mA cm^−2^	2018 (ref. 209)
SnSe nanosheets/rGO (photoanode)	Hydrothermal	ITO	350 W Xe arc lamp (100 mW cm^−2^)	0.5 M Na_2_SO_4_	0.8 V_Ag/AgCl_	0.064 mA cm^−2^	2017 (ref. 217)
Graphene/CoSe or NiSe (photoanode or photocathode)	CVD and electrodeposition	Ni mesh	350 W Xe arc lamp (100 mW cm^−2^)	1 M KOH	0 V_Ag/AgCl_	3.0 mA cm^−2^	2016 (ref. 218)

a Gr = graphene; GNWs = graphene nanowalls; NSs = nanosheets; NTs = nanotubes; NWs = nanowires; LAL = laser ablation in liquid; SILAR = successive ionic layer adsorption reaction; GCE = glassy carbon electrode; g-C_3_N_4_ = graphitic carbon nitride.

Transition metal selenides are also considered good candidates for PEC water splitting. These materials have a similar structure and properties that the transition metal sulfides. They not only share some similarities but also differences. For example, the transition metal selenides have better electrical conductivity than the transition metal sulfides.^206^ However, these materials are also vulnerable to recombination in PEC systems.^160^ There are few studies about the use transition metal selenides/graphene (and its derivatives) hybrids as PEC electrodes.^207^ Several roles of graphene (and its derivatives) have also been emphasized such as charge acceptors and separators or as protective, catalytic and supporting layers.^208^ In other case, graphene (and its derivatives) sheets can act as conductive scaffold which enhances the charge carrier transfer, reducing both charge transfer resistance and carrier recombination.^192,209–211^ Interestingly, transition metal selenides have shown better responses compared to the sulfides (Table 4), representing a new opportunity to be explored. An important advance on the field was the outstanding performance of Cu(In,Ga)Se_2_ (CIGS) photocathodes after rGO modification, in which rGO as protective and binder layers improves the photocurrent density and stability.^211^

In summary, among the methodologies, the chemical methods (*e.g.* hydrothermal and solvothermal) have been more used to synthesize graphene-based TMCs nanocomposites. These one-step methods produce nanoparticles with defined morphology and homogeneously distributed on the surface of the rGO sheets. Although these methods produce rGO sheets with structural defects and residual functional groups, their attributes are still comparable to graphene sheets. These residual oxygen functionalities facilitate better attachment of nanoparticles to the rGO sheets.^212^ Besides, these methods can achieve high-yield, large-scale and low-cost production.^66^ Graphene and its derivatives as protective layers have also been reported in the PEC systems.^183^ Among the materials, SLG is the most used as protective layers because it has much less defects and less functional groups on surface and edges. CVD is one of the techniques that has been most used to synthesize SLG sheets in a more reproductive way, with well-known composition and more accurate properties. However, expensive and tedious procedures and inability to accomplish large scale production limit commercial deployment.^200,212^ When rGO acts as protective layer, the type and residual amount of functional groups on the rGO surface must be controlled by selecting various oxidation and reduction agents, as well as adjusting the reduction time.^211^

Although graphene-based TMCs have been widely used in many PEC systems, there are few studies on the interface between these semiconductors and the graphene sheets. This understanding will be essential to guide the design of novel graphene hybrids with controllable chemical compositions, spatial distributions and desired structures. The development of density functional theory (DFT) calculations and *in situ* techniques will also be fundamental to provide more information about the surface chemical and structural evolution of these photoelectrodes during PEC water splitting processes. The improvement of the PEC stability of graphene-based TMCs is also another challenge.^213^

### 3.2 Graphene-coated silicon photoelectrodes

**Fig. 9:**
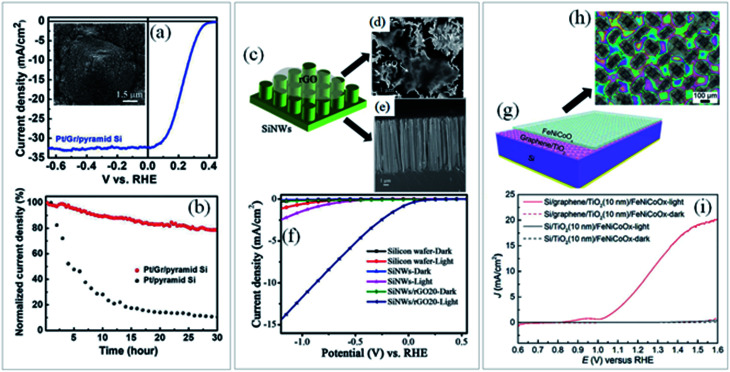
(a) Photocurrent density–potential curves of Pt/Gr/pyramid Si photocathode measured in 1 M HClO_4_ electrolyte under simulated illumination (AM 1.5G). The inset shows the SEM image of Pt NPs depositing on the surface of graphene/pyramid Si photocathode. (b) The stability test of the Pt/pristine pyramid Si and Pt/graphene/pyramid Si Schottky junction photocathode under illumination at a voltage of −0.3 V_RHE_. Reprinted with permission from ref. 41 Copyright 2019, Wiley-VCH. (c) Schematic illustration, (d) cross-section and (e) top view FESEM images for SiNWs/rGO20 composite. (f) Photocurrent density–potential curves of polished silicon wafer, SiNWs and SiNWs/rGO20 photocathodes measured in 0.1 M H_2_SO_4_ and 0.5 M K_2_SO_4_ electrolyte in the dark and under simulated illumination (AM 1.5G). Reprinted with permission from ref. 229 Copyright 2016, Royal Society of Chemistry. (g) Schematic structure and (h) optical image of the Si/Gr/TiO_2_/FeNiCoO_*x*_ structure. (i) Photocurrent density–potential curves of Si/TiO_2_/FeNiCoO_*x*_ and Si/graphene/TiO_2_/FeNiCoO_*x*_ photoanodes measured in 1 M NaOH electrolyte in the dark and under simulated illumination (AM 1.5G). Reprinted with permission from ref. 230 Copyright 2018, Royal Society of Chemistry.

Being an earth-abundant material, silicon (Si) is one of the most studied semiconductors in the field of PEC water splitting as a light absorber thanks to its suitable band gap (1.12 eV).^219^ Its CB edge position (−0.5 V_NHE_ at pH = 0) is sufficiently more negative than the reduction potential of protons for hydrogen production (H^+^/H_2_).^185^ This semiconductor shows a great potential for solar hydrogen production. However, there are some limitations of utilizing Si as photoelectrodes in a PEC cell. Because of large reflection of conventional planar Si wafers, various textured Si based photoelectrodes has been studied, such as Si nanowires array,^220^ Si pyramid array^221^ and Si nanopore array.^222^ The increased surface area in these textured photoelectrodes causes undesirable charge carrier recombination and accelerates surface photocorrosion/passivation during the electrochemical reactions.^219^ An alternative approach to reduce this problem is the use of suitable protective layers on the electrode. Due to their high optical transmittance, chemical inertness, good electrical conductivity and large surface area, graphene and its derivatives can be easily applied to semiconductor surfaces over large areas.^223^ These materials, specifically SLG, can be used as protective coating layer to improve the photocorrosion/passivation resistance of Si-based photoelectrodes.^223^ Among the techniques employed, CVD is one of the most used to synthesize SLG sheets on the Si photoelectrodes and their high PEC performances are summarized in Table 5. The formation of pyramid-like Si/graphene Schottky junctions with a 3D architecture (Fig. 9a) showed to be a promising approach to improve the performance and durability of Si-based PEC systems for water splitting^41^ and reflect the excellent role of SLG as passivation layer which remains a relatively stable current during 30 h (Fig. 9b). Some reports related that the PEC performance of graphene-coated Si photoelectrodes is directly dependent on the number of graphene layers.^224^ The surface modification with layered graphene changes band bending of the Si surface and affects the kinetics of hydrogen production.

**Table 5:** PEC performances of graphene-coated silicon photoelectrodes^a^

Photoelectrodes (photoanode or photocathode)	Preparation method	Light source	Electrolyte	Onset potential	Applied potential	Photocurrent density	Year (ref.)
Si/double layer Gr (photocathode)	CVD, LbL transfer and plasma treatment	300 W Xe arc lamp (AM 1.5G, 100 mW cm^−2^)	1 M HClO_4_	0.20 V_RHE_	−0.7 V_RHE_	−30 mA cm^−2^	2017 (ref. 225)
Si NWs/rGO (photocathode)	MCECE	300 W Xe arc lamp (AM 1.5G, 100 mW cm^−2^)	1 M HClO_4_	0.33 V_RHE_	0 V_RHE_	−23.1 mA cm^−2^	2018 (ref. 226)
Si/hydrophobic rGO (photocathode)	Atmospheric plasma CVD	300 W Xe arc lamp (AM 1.5G, 100 mW cm^−2^)	1 M HClO_4_	−0.15 V_RHE_	−0.6 V_RHE_	−3.3 mA cm^−2^	2020 (ref. 227)
Si/multi-layer Gr (photocathode)	APCVD	300 W Xe arc lamp (AM 1.5G, 100 mW cm^−2^)	1 M H_2_SO_4_	−0.36 V_RHE_	−0.385 V_RHE_	−28.3 mA cm^−1^	2019 (ref. 224)
Si/GNWs/CdTe (photoanode)	RF-PECVD and VTD	500 W Xe arc lamp (100 mW cm^−2^)	0.5 M Na_2_S	—	0 V_RHE_	3.0 mA cm^−2^	2017 (ref. 228)
Si/Gr/GaN NWs (photoanode)	CVD and MBE	500 W Xe arc lamp (100 mW cm^−2^)	1 M NaOH	—	0 V_Ag/AgCl_	0.24 mA cm^−2^	2017 (ref. 24)

a Gr = graphene; GNWs = graphene nanowalls; LbL = layer by layer; APCVD = atmospheric pressure chemical vapor deposition; ALD = atom layer deposition; LPCVD = low pressure chemical vapor deposition; MCECE = metal-catalysed electroless chemical etching; RF-PECVD = radio frequency plasma enhanced chemical vapor deposition; VTD = vapor transport deposition; MBE = molecular beam epitaxy.

Graphene and its derivatives could also be used as catalysts for a solar-driven hydrogen evolution reaction on Si-photocathodes. Fig. 9c–f displayed a schematic illustration, SEM images and PEC behaviour of Si nanowires (NWs) wafer covered with rGO and obtained by a facile and controllable electrochemical method.^229^ In these circumstances, rGO acts as an acceptor of the electrons generated in the Si NWs photocathode that improves the separation of the photogenerated charge carriers. Consequently, the enhancement of the photoelectrochemical performance of the SiNWs/rGO20 photocathode may be greatly attributed to the high electrical conductivity and the boosted charge transfer rate.

Generally, an integrated Si photoelectrode system for PEC water splitting requires a Si absorber, a protective layer and a catalytic layer.^219^Fig. 9g and i, for example, illustrate the schematic structure, the optical image and PEC performance of the Si/graphene/TiO_2_/FeNiCoO_*x*_ photoanode.^230^ The role of the rGO in the photoelectrode was attributed to the efficient charge separation at the interface (Si/graphene) and the defects in the graphene layer that provided electrochemically active sites for water oxidation. Other records also emphasize that the changes of the surface functional groups on graphene and its derivatives^227^ and the different graphene deposition techniques on the Si substrate^224,226^ can significantly influence the performance of PEC water splitting.

**Fig. 10:**
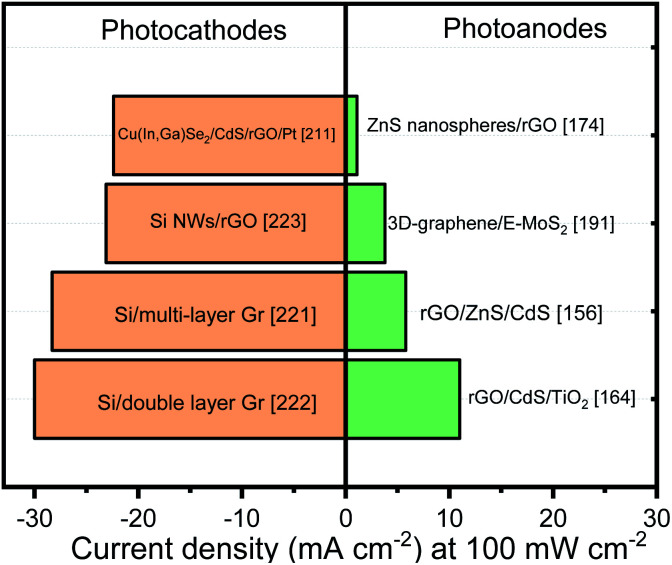
Photocurrent benchmark of graphene/non-oxides photoelectrodes. Data were extracted from various reports in the literature.

Fig. 10 displayed the best photocurrent performance of non-oxides photoelectrodes. Gr/Si-based photocathodes have the best photoresponse due to Si excellent properties as absorber where Gr or rGO is mainly used as protective layer in order to improve the photocathode stability during operation. However, techniques used to deposit the graphene layer are expensive which limits their scale-up. On the other hand, a complex system formed by a combination of TMCs and rGO has shown a promising and stable performance during operation as photocathode, but the use of Pt compromises its deployment. In addition, although graphene can catalyze the HER, the results are not satisfactory yet and new strategies need to be developed in the photocathodes design.

In contrast, rGO/sulfide photoanodes synthesized by solvothermal methodologies have shown good PEC response. For these systems, graphene (and its derivatives) is employed not only as an electron acceptor and transporter but also as growth support, allowing the formation of optimized structures. As it can be noticed, the systems that have raised the maximal current are graphene/heterojunction nanocomposites, indicating that graphene by itself does not resolve the photoelectrodes limitations.

In case on non-oxide photoelectrodes, the incorporation of graphene sheets in the semiconductor matrix increases the photocurrent density value, without changing the *E*_onset_. Furthermore, some factors directly affect the final performance of these photoelectrodes, including synthesis methodology, graphene content in the composite, semiconductor morphology, interfacial interaction and contact area between graphene and semiconductor.^42^

## 4 Final remarks and perspectives

The introduction of graphene in semiconducting photoelectrodes aiming the production of hydrogen and oxygen has become a potential strategy to design the next generation of photocatalysts. In this review, we have summarized the most recent accomplishments where graphene structures were combined with different semiconductor electrodes (metal oxides and non-metal oxides) in PEC applications. In all cases discussed, the insertion of graphene or other graphene derivatives has always led to an improvement in the performance and/or stability of the electrode. Regardless the positive and synergic effects, the mechanisms that drive the charge carrier dynamics within the system and how they operate are not fully understood, bringing new opportunities to be explored in the field.

In view of charge carrier dynamics, graphene and its derivatives addition led to a more efficient charge transport through their layers compared to the photoelectrodes where grain boundaries and other defects act as charge carrier traps are present. Although for a single layer graphene, both electron and hole transport are possible and efficient, most samples are not pure or single layer graphene. Graphene oxide, doped graphene, reduced graphene oxide and other derivatives tend to benefit of one carrier to another. And even applying sophisticated techniques, it is hard to separate the effect on the electron or hole dynamics.

Many reports have demonstrated that graphene can also passivate the surface states in oxides and non-oxides photoelectrodes leading a significant enhancement performance and stability. Here, passivation also comes from the possibility to modify graphene edges and basal planes. Nevertheless, even if graphene has shown remarkable performance as protective layer, some reports has shown that the deposition methodologies bring inherent problems to accomplish this role.

Among graphene derivatives, rGO is the most employed probably associated with its facile synthesis and low-cost precursors. As graphene, rGO can acts as a passivation layer, excels the photoexcited charge carrier separation and, consequently, reduce their recombination at specific points of the electrode. An interesting application on rGO widely reported for non-oxide photoelectrodes consist in using it as growth supporting to create ordered structures with improved properties.

Incorporating GQDs on metal oxide photoelectrodes represents a promising and not extensively studied field. In addition to the charge separation/transport and stability gains, the coupled system formed by the photoelectrodes and GQDs also showed a superior light absorption compared to the non-coupled electrode which can be related to a higher photocurrent value. However, the amount of GQDs needs to be carefully controlled since this excess block the active sites and inhibits the OER/HER at the surface. In fact, this is an issue commonly found for other carbonaceous materials when incorporated in the electrodes.

Despite some studies present promising results in the field, some issues are still elusive; several reports affirm that a Schottky junction is formed between the semiconductor and the graphene but, to our knowledge, the junction nature between the semiconductors and graphene is still under debate and not fully understood. Otherwise, the usage of *in situ*/*operando* techniques to characterize graphene-bases photocatalysts is extremely valuable because they can provide fundamental aspects for sharpening our understanding on the nature of active sites, reaction mechanisms, and charge carrier dynamics. These techniques could also assist us to elucidate the chemical states and electronic structures, especially at the interface semiconductor–electrolyte.

Notwithstanding all the advantages presented in this review, it is important to point that better strategies to synthesize graphene coupled to photoelectrodes need to be developed. Despite all methodologies reported, the desired performance depends on a successful graphene addition or transfer to the substrate. For this reason, surface engineering plays an important role to further improve the PEC systems for water splitting.

The path ahead for graphene and its derivatives in photoelectrodes towards water splitting reactions is bright, but challenging, and many efforts in the chemistry and physics of these systems are still needed. The full understanding of how graphene operates may help us to tailor the properties of the semiconducting materials, to adjust the synergy and to gain benefits from all parts. We believe that for many years it will be a fascinating research area because of the many possibilities and opportunities.

## Conflicts of interest

There are no conflicts to declare.

## Supplementary Material
